# Supplementary material for: CryoDataBot: a pipeline to curate cryoEM datasets for AI-driven structural biology
Source: Gigascience. 2025 Oct 22;14:giaf127. doi: 10.1093/gigascience/giaf127 (PMC12596181; doi:10.1093/gigascience/giaf127)
Supplement: giaf127_Supplement_figures [file giaf127_supplement_figures.pdf]

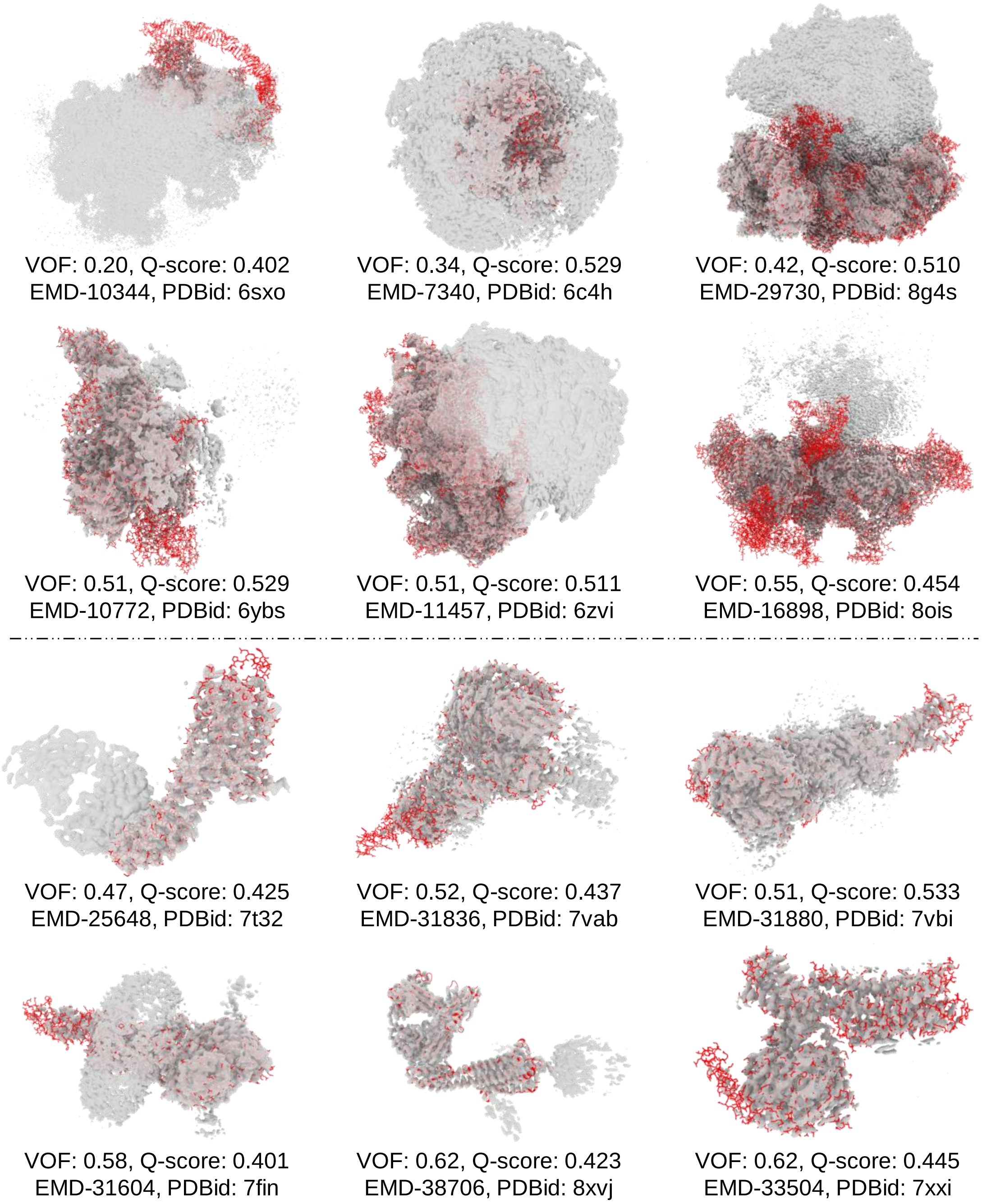

Fig. S1 Examples discarded during MMF validation (low VOF score despite good Q-score). The top six examples are selected from 71 entries excluded during the construction of the ribosome experimental dataset, while the bottom six are drawn from 6 entries excluded during the construction of the G protein experimental dataset. The VOF score effectively captures global inconsistencies that may not be detected by Q-score alone.

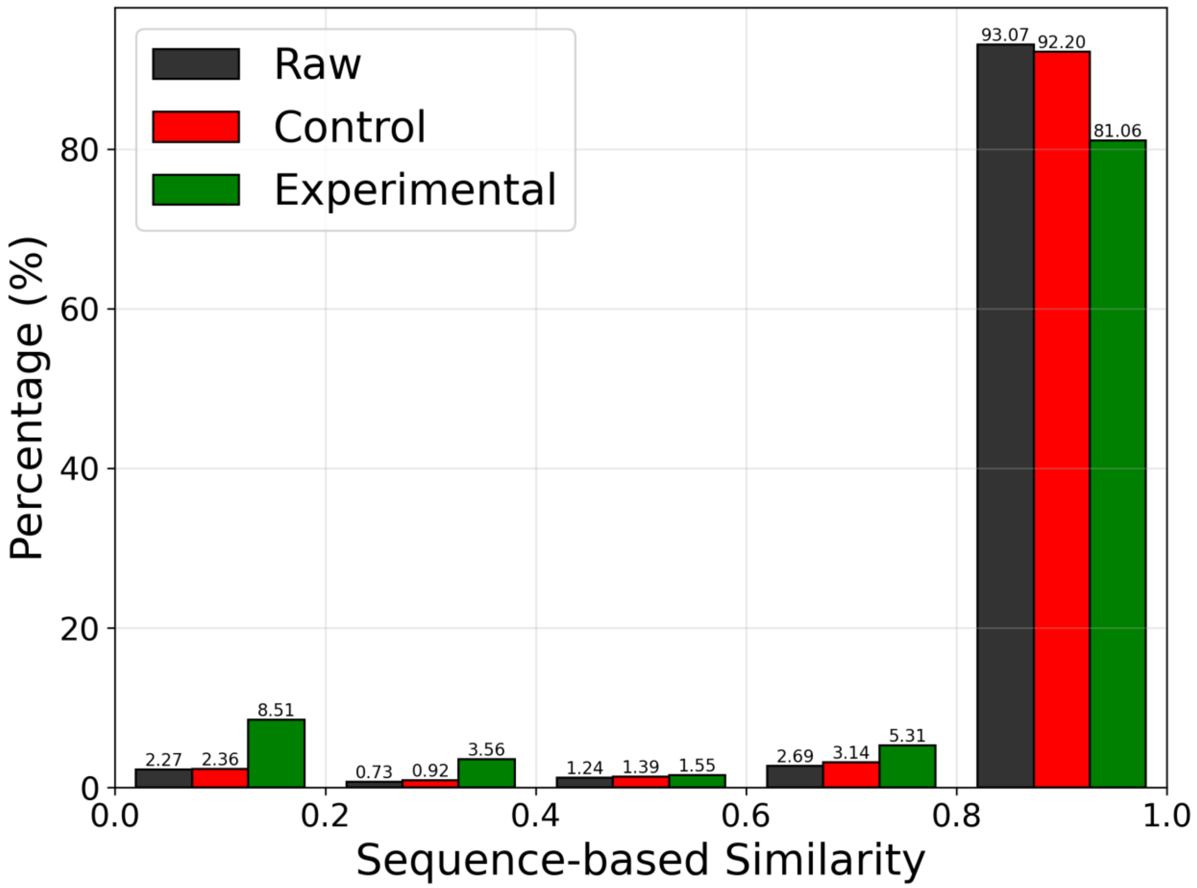

Fig. S2 Comparative analysis of sequence similarity across datasets. For each entry **X** (containing *x* chains), each chain was compared via BLASTp against every chain from all other entries. If another entry **Y** (containing *y* chains) has *n* chains identified as homologous (e-value < 0.001 from the BLASTp results) to any chains in **X**, the sequence-based similarity **Y** to **X** is defined as *n/y*. This asymmetric scoring yields $N*(N–1)$ pairwise similarity scores for a dataset with *N* entries. A larger proportion of high scores indicates greater sequence-level redundancy. As shown, the Raw and Control datasets exhibit comparable redundancy, whereas the Experimental dataset shows a marked reduction—consistent with the structural similarity trends in Fig. 2b.

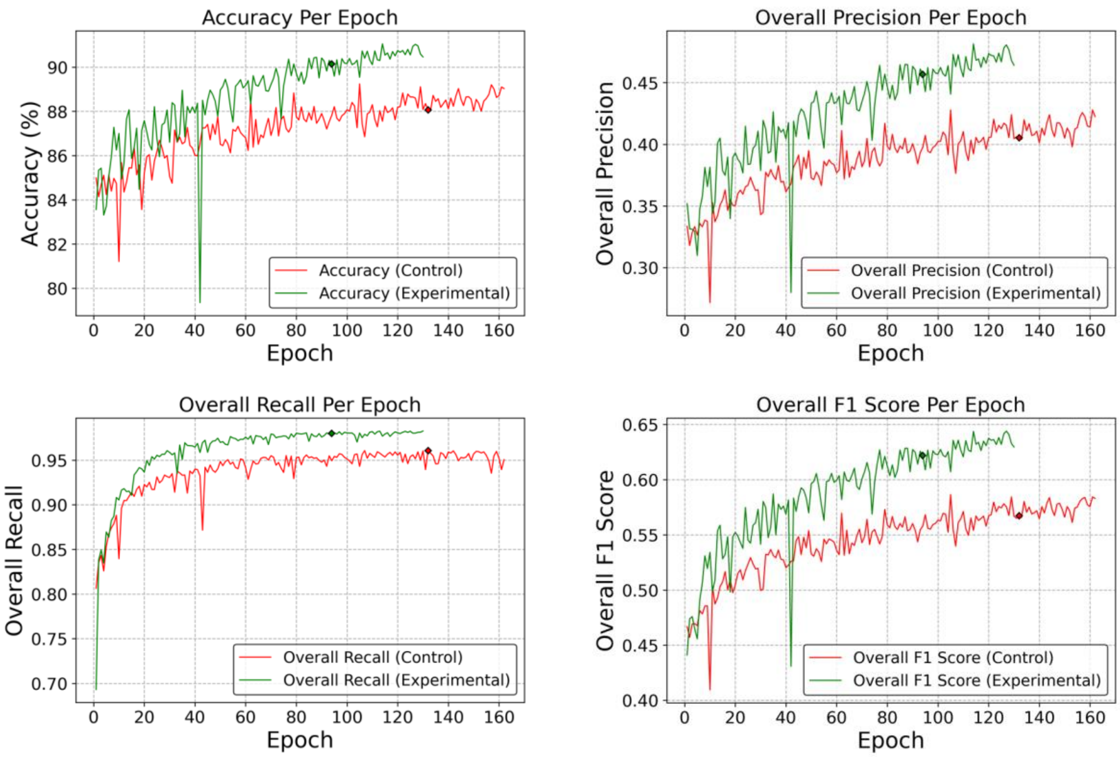

Fig. S3 Training dynamics of the U-Net models on control and experimental datasets. Evolution of overall accuracy, precision, recall, and F1 score on the validation set during training of the control model (red) and the experimental model (green). Epochs corresponding to peak performance are marked by diamonds at epoch 132 (control) and epoch 94 (experimental). The experimental model achieved higher validation metrics in fewer epochs, indicating improved training efficiency.

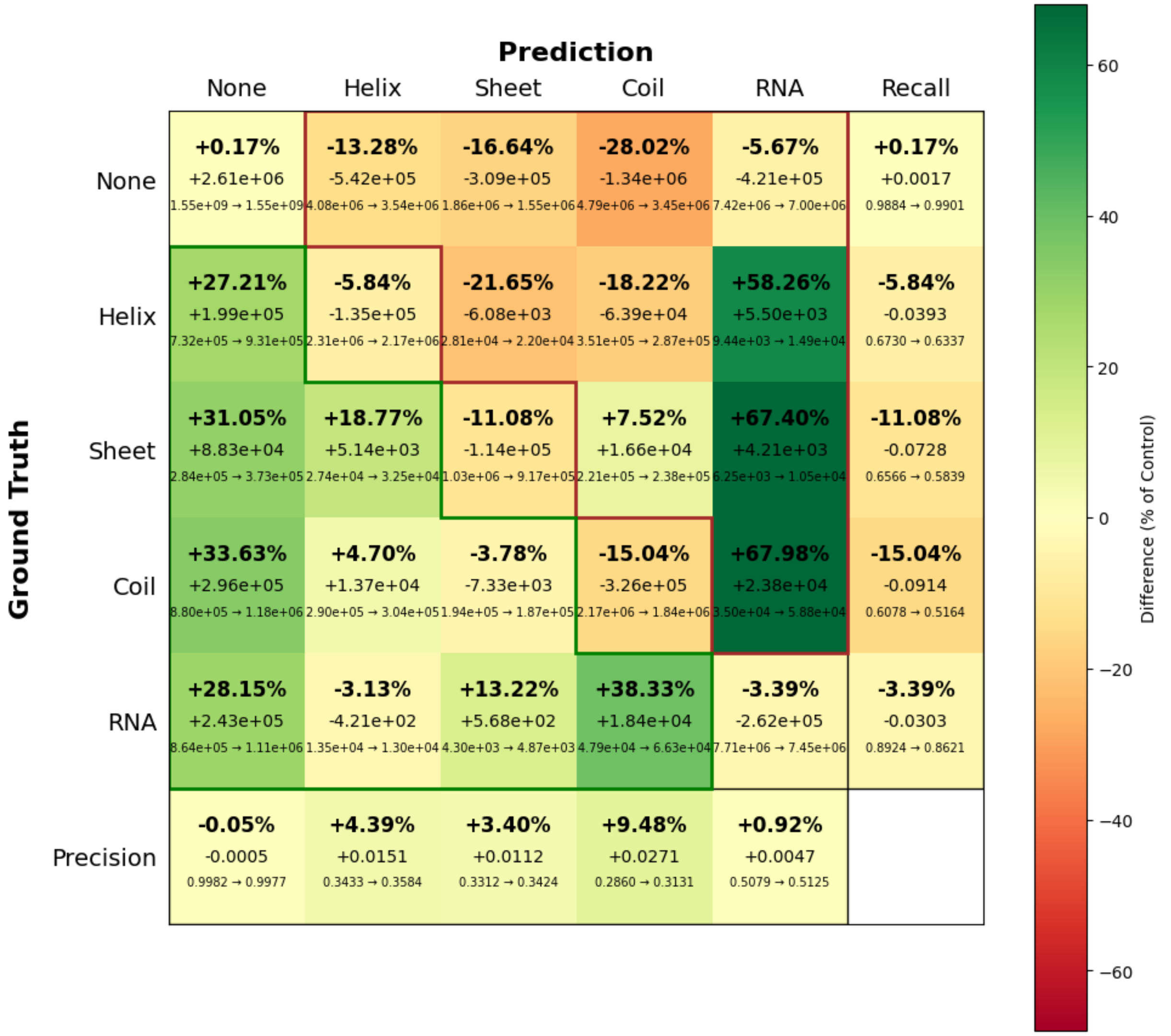

Fig. S4 Comparison of confusion matrices between the control and experimental models for secondary structure prediction, including recall and precision metrics. Each cell displays three lines: (bottom) control model count → experimental model count, (middle) the absolute difference (experimental minus control), and (top) the ratio of difference to control. Cells are color-coded based on this ratio. Brown outlines indicate false positives (FP), relevant to precision calculation, while green outlines indicate false negatives (FN), relevant to recall calculation.

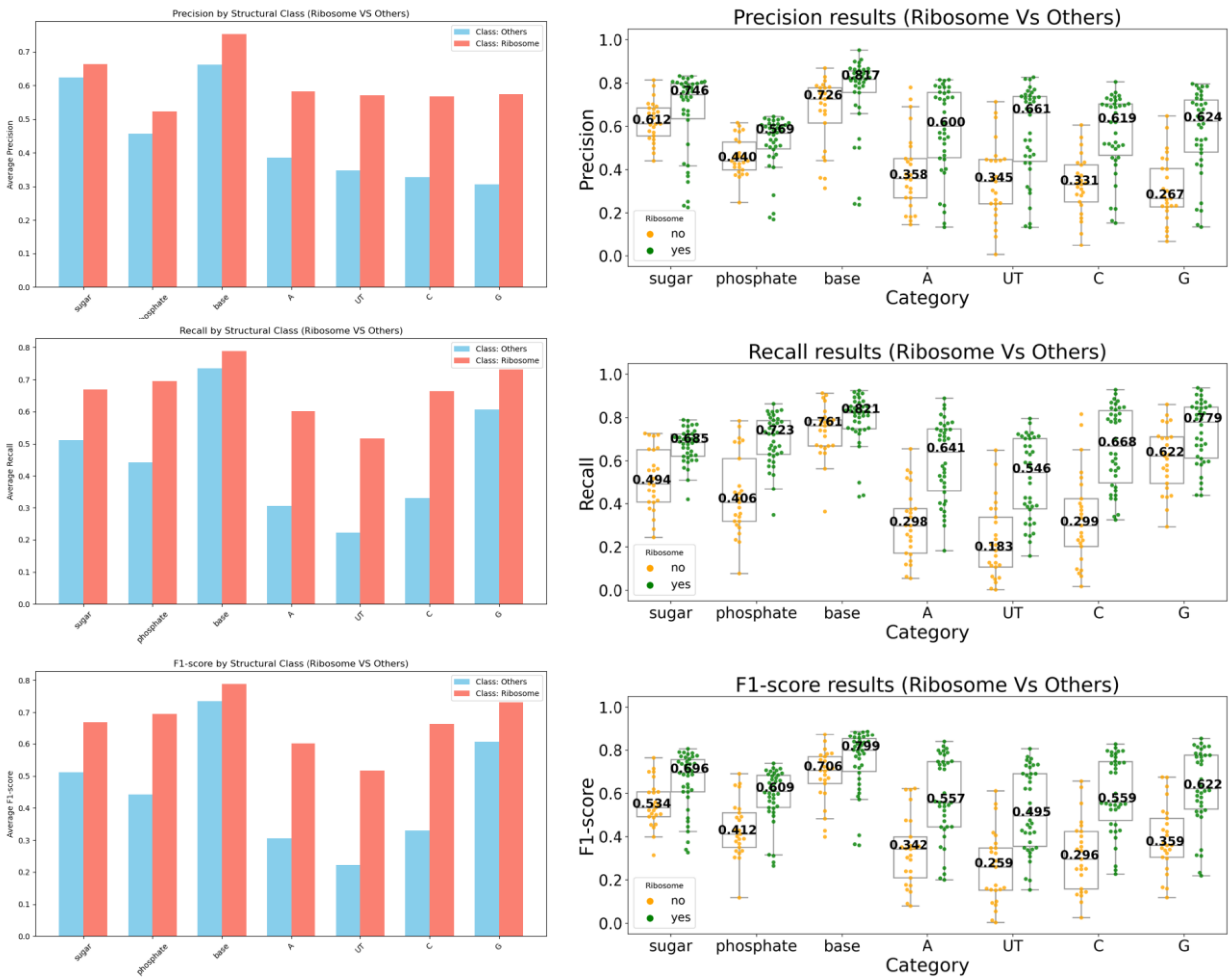

Fig. S5 Comparison of Precision, Recall and F1-scores for retrained CryoREAD with CryoDataBot training set showing the difference in performance on ribosomes and other classes in the test set. The bar charts show average precision, recall, and F1 scores for ribosomes (38 cases) and other classes (25 cases). The box plots show the individual data points representing EMDB maps along with the medians annotated. Since the retrained CryoREAD with CryoDataBot dataset was trained with exclusively ribosome-based data, the performance on the test set is affected by it. For all 7 structural classes (Sugar, Phosphate, Base, A, U/T, C, G) the metric values for ribosome test cases are higher than those on other classes.

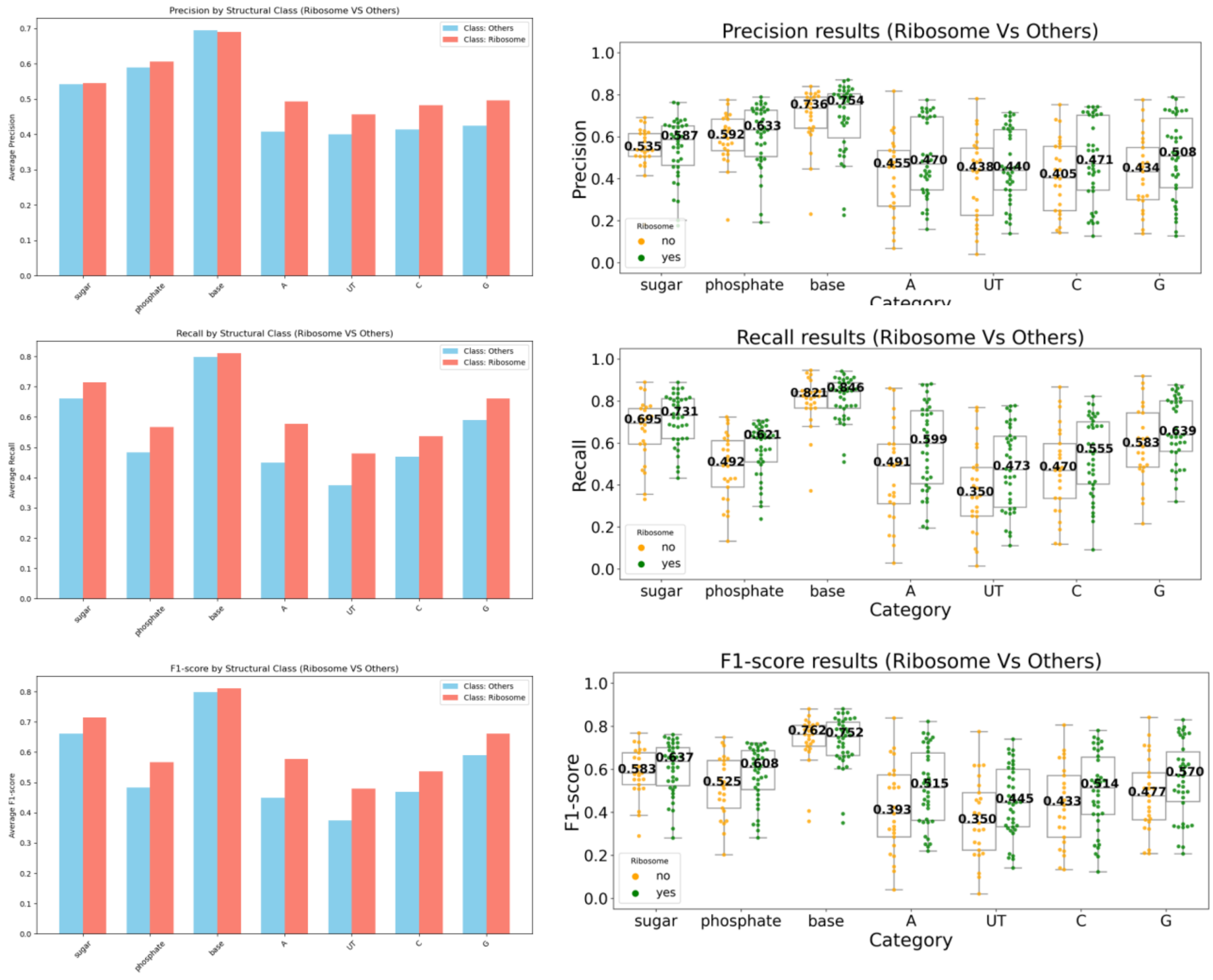

Fig. S6 Comparison of Precision, Recall and F1-scores for original CryoREAD showing the difference in performance on ribosomes and other classes in the test set. The bar charts show average precision, recall, and F1 scores for ribosomes (38 cases) and other classes (25 cases). The box plots show the individual data points representing EMDB maps along with the medians annotated. The original CryoREAD was trained with different classes of RNA and DNA. Contrary to Fig. S5, the difference in performance for ribosome test sets and other classes of RNA/DNA is less. The average base precision is higher for other RNA/DNA classes than for ribosomes. The box plot for F1-scores also shows that the median F1-score for other classes is higher than those with ribosome test cases.

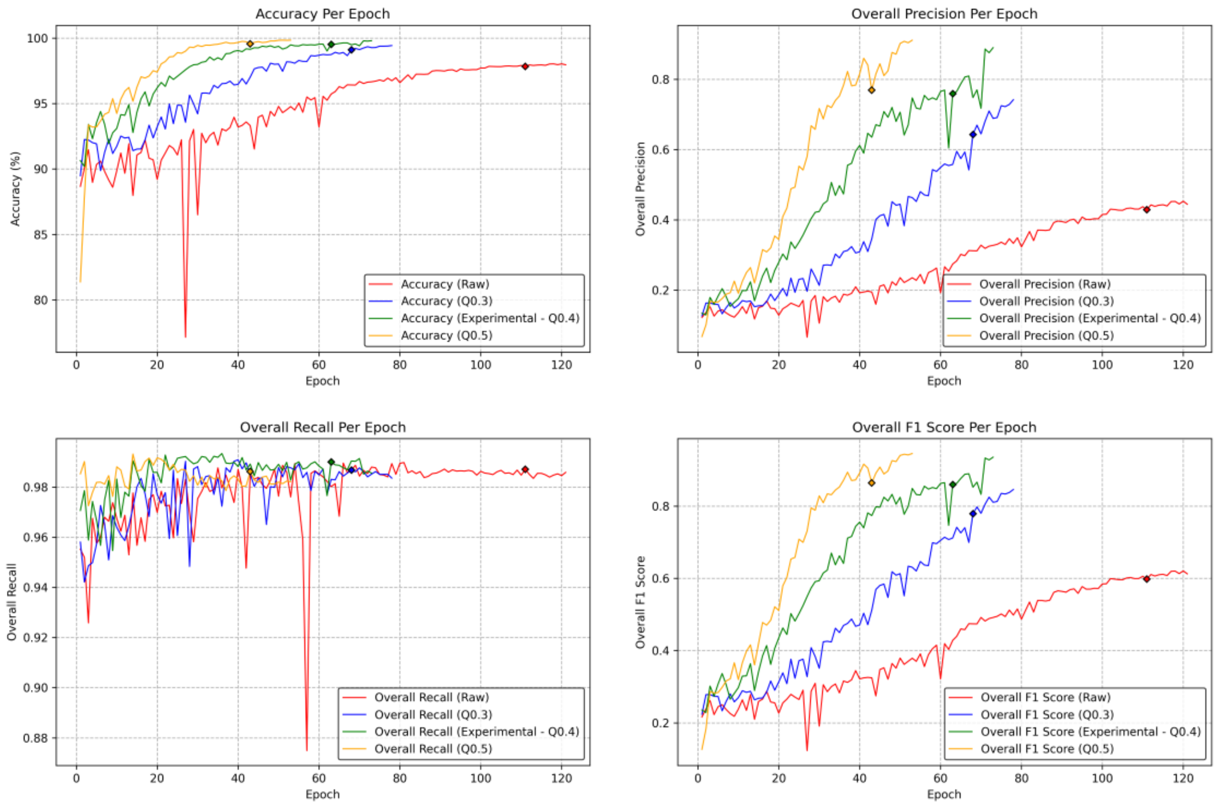

Fig. S7 Training dynamics of U-Net models training on G protein datasets. Evolution of overall accuracy, precision, recall, and F1 score on the validation set during training of the raw (red), Q0.3 (blue), experimental (green) and Q0.5 (orange) models. Epochs corresponding to the best performance are marked by diamonds at epoch 111 (raw), 68 (Q0.3), 63 (experimental - Q0.4), and 43 (Q0.5). Compared to the raw model, the experimental model reached higher validation metrics in fewer epochs, indicating improved training efficiency.
