# Supplementary material for: CryoDataBot: a pipeline to curate cryoEM datasets for AI-driven structural biology
Source: Gigascience. 2025 Oct 22;14:giaf127. doi: 10.1093/gigascience/giaf127 (PMC12596181; doi:10.1093/gigascience/giaf127)
Supplement: giaf127_GIGA-D-25-00271_Original_Submission [file giaf127_giga-d-25-00271_original_submission.pdf]

GigaScience

CryoDataBot: a pipeline to curate cryoEM datasets for AI-driven structural biology

--Manuscript Draft--

**Manuscript Number:**	GIGA-D-25-00271
**Full Title:**	CryoDataBot: a pipeline to curate cryoEM datasets for AI-driven structural biology
**Article Type:**	Research
**Funding Information:**	National Institute of General Medical Sciences (R01GM071940) — Professor Z. Hong Zhou<br>NIGMS (R01GM133840) — Prof Daisuke Kihara<br>NSF (IIS2211598) — Prof Daisuke Kihara
**Abstract:**	Cryogenic electron microscopy (cryoEM) has revolutionized structural biology by enabling atomic-resolution visualization of biomacromolecules in near-native states. To automate the labor-intensive process of atomic model building from cryoEM maps, artificial intelligence (AI) methods have emerged as powerful tools. However, the development and benchmarking of AI-based automated modeling tools have been hindered by the lack of high-quality, standardized datasets. We present CryoDataBot, a user-friendly, automated pipeline that addresses this gap. It streamlines data retrieval, preprocessing, and labeling, with fine-grained quality control and flexible options for diverse research needs, enabling efficient generation of robust datasets. CryoDataBot's effectiveness is demonstrated through improved training efficiency in U-Net models and rapid, successful retraining of CryoREAD, a widely used automated RNA modeling tool. By standardizing the workflow and minimizing user input, CryoDataBot improves reproducibility and reduces technical barriers. It empowers researchers to advance AI-driven structural biology and supports broad applications in model training, validation, and structural analysis.
**Corresponding Author:**	Z. Hong Zhou<br>UCLA: University of California Los Angeles<br>Los Angeles, California UNITED STATES
**Corresponding Author Secondary Information:**	
**Corresponding Author's Institution:**	UCLA: University of California Los Angeles
**Corresponding Author's Secondary Institution:**	
**First Author:**	Z. Hong Zhou
**First Author Secondary Information:**	
**Order of Authors:**	Z. Hong Zhou<br>Qibo Xu, PhD<br>Leon Wu<br>Michael Rebelo<br>Shi Feng<br>Xinye Yu<br>Harhana Harheen<br>Daisuke Kihara
**Order of Authors Secondary Information:**	
**Additional Information:**	
**Question**	**Response**

Powered by Editorial Manager® and ProduXion Manager® from Aries Systems Corporation

Are you submitting this manuscript to a special series or article collection?	No
**Experimental design and statistics**<br><br>Full details of the experimental design and statistical methods used should be given in the Methods section, as detailed in our Minimum Standards Reporting Checklist. Information essential to interpreting the data presented should be made available in the figure legends.<br><br>Have you included all the information requested in your manuscript?	Yes
**Resources**<br><br>A description of all resources used, including antibodies, cell lines, animals and software tools, with enough information to allow them to be uniquely identified, should be included in the Methods section. Authors are strongly encouraged to cite Research Resource Identifiers (RRIDs) for antibodies, model organisms and tools, where possible.<br><br>Have you included the information requested as detailed in our Minimum Standards Reporting Checklist?	Yes
**Availability of data and materials**<br><br>All datasets and code on which the conclusions of the paper rely must be either included in your submission or deposited in publicly available repositories (where available and ethically appropriate), referencing such data using a unique identifier in the references and in the "Availability of Data and Materials" section of your manuscript.<br><br>Have you have met the above requirement as detailed in our Minimum	Yes

Standards Reporting Checklist?	
GigaScience has policies and guidelines in place for the use of generative AI-writing tools such as ChatGPT. If you have used such writing tools to assist with writing the manuscript this must be declared and cited in the text. Authors should not list AI-writing tools and other AI-assisted technologies as an author or co-author and should acknowledge that they are fully responsible for text generated or refined by AI-writing tools.<p><br><br>A summary of use (particularly in the introduction or among methods) needs to be included at the end of the paper, and the outputs should also be included as a supplementary file hosted in GigaDB or other open repositories. Please read our guidelines for more information. <p><br><br>By submitting to GigaScience, you are aware of the journal's AI-writing tools policy, and if you have declared use of such tools below, you have acknowledged this where appropriate in your manuscript and have made a summary of use and outputs available. <p><br><b>AI-assisted writing tools have been used in the preparation of this manuscript?	No

Powered by Editorial Manager® and ProduXion Manager® from Aries Systems Corporation

Manuscript with figures and SI

Click here to access/download;Manuscript;CryoDataBot-manuscript-withAllFigures-and-SI-July7-2025-Zhou.pdf

CryoDataBot: a pipeline to curate cryoEM datasets for AI-driven structural biology

Qibo Xu[1,2], Leon Wu[1,3], Michael Rebelo[1,4], Shi Feng[1,3], Xinye Yu[1,2], Farhanaz Farheen[5], Daisuke Kihara[5,6], Z. Hong Zhou[1,2,3,4,*]

[1]California NanoSystems Institute, University of California, Los Angeles, CA 90095, USA

[2]Department of Microbiology, Immunology, and Molecular Genetics, University of California, Los Angeles, CA 90095, USA

[3]Department of Bioengineering, University of California, Los Angeles, CA 90095, USA

[4]Department of Chemistry and Biochemistry, University of California, Los Angeles, CA 90095, USA

[5]Department of Computer Science, Purdue University, West Lafayette, IN 47907, USA

[6]Department of Biological Sciences, Purdue University, West Lafayette, IN 47907, USA

[*] Correspondence should be addressed to Z.H.Z. (Hong.Zhou@UCLA.edu)

Abstract

Cryogenic electron microscopy (cryoEM) has revolutionized structural biology by enabling atomic-resolution visualization of biomacromolecules in near-native states. To automate the labor-intensive process of atomic model building from cryoEM maps, artificial intelligence (AI) methods have emerged as powerful tools. However, the development and benchmarking of AI-based automated modeling tools have been hindered by the lack of high-quality, standardized datasets. We present CryoDataBot, a user-friendly, automated pipeline that addresses this gap. It streamlines data retrieval, preprocessing, and labeling, with fine-grained quality control and flexible options for diverse research needs, enabling efficient generation of robust datasets. CryoDataBot's effectiveness is demonstrated through improved training efficiency in U-Net models and rapid, successful retraining of CryoREAD, a widely used automated RNA modeling tool. By standardizing the workflow and minimizing user input, CryoDataBot improves reproducibility and reduces technical barriers. It empowers researchers to advance AI-driven structural biology and supports broad applications in model training, validation, and structural analysis.

Introduction

Accurately determining the three-dimensional structure of biomacromolecules is fundamental to understanding their function and guiding therapeutic development[1–6]. Traditional approaches like X-ray crystallography and nuclear magnetic resonance (NMR) spectroscopy have long been central to structural biology[7–10]. However, these methods are often limited by poor protein solubility, sample heterogeneity, and intrinsic resolution constraints. Furthermore, they typically require considerable manual effort, such as sample crystallization. In contrast, cryogenic electron microscopy (cryoEM) enables high-resolution imaging of large and heterogeneous macromolecular complexes in their native conformations[11–18]. This capability allows cryoEM to reveal intricate structural details across diverse conformational states under physiological conditions.

Despite substantial advancements in cryoEM instrumentation and image processing[19–24], structure modeling remains a major bottleneck. Like crystallography and NMR, deriving atomic models from cryoEM data can take weeks to months and remains error-prone[25], even with

sophisticated tools such as PHENIX[26], Coot[27] and ChimeraX[28]. As cryoEM continues to expand in scope and target increasingly complex biomacromolecules, recent research has turned to artificial intelligence (AI) for optimizing EM maps and automating modeling[29–37]. These methods often employ architectures such as convolutional neural networks, graph neural networks, and U-Net models[38–40]. Regardless of implementation, these approaches critically depend on the availability of high-quality training datasets characterized by structural diversity, accurate labels, and low redundancy[41,42].

AI-based modeling tools such as DeepTracer[29], ModelAngelo[30], and CryoREAD[31] typically build training datasets by retrieving cryoEM maps and associated atomic models from the Electron Microscopy Data Bank (EMDB)[43] and the Protein Data Bank (PDB)[44]. These maps are then resampled to a uniform voxel size, and structural labels are generated from the atomic coordinates. The data, comprising resampled maps and labels, is partitioned into smaller 3D sub-volumes to support efficient model training. Some pipelines include additional quality enhancement steps, such as map–model fitness (MMF) evaluation and redundancy handling (Table 1). While these practices improve dataset fidelity and modeling accuracy, the absence of standardized construction pipelines across tools impedes reproducibility and complicates cross-comparisons. Moreover, the processed datasets are generally not publicly available, presenting a major hurdle for new developers who must rebuild training data from scratch.

A recent effort, Cryo2StructData[45], began to address these issues by releasing a large-scale, publicly accessible cryoEM dataset with standardized features and curated labels. However, it omits key quality control steps, including MMF evaluation and redundancy handling (Table 1), both essential for producing consistent datasets and optimizing model training. Furthermore, it provides only voxel-resampled maps at a fixed resolution of 1.0 Å, which are not suitable for all AI architectures (e.g., DeepTracer uses 0.5 Å[29]; ModelAngelo uses 1.5 Å[30]). These limitations underscore the need for a flexible, integrated framework capable of generating high-quality, customizable cryoEM training datasets with built-in quality control and atom-level labeling.

To address this gap, we have developed CryoDataBot, a user-friendly, GUI-based data generation pipeline that empowers researchers—regardless of computational expertise—to create tailored, high-quality cryoEM datasets with just a few inputs. CryoDataBot supports filtering

based on user-defined parameters, including residue-level similarity, Q-scores[46], MMF validation thresholds, and any available EMDB metadata filters[47]. By providing a standardized and modular framework, CryoDataBot simplifies dataset construction, facilitates targeted model training, and promotes reproducibility and benchmarking in AI-driven structural biology research.

Results

Pipeline of CryoDataBot and Functionality of Each Module

We design CryoDataBot as a tool to automate generation of high-quality datasets for AI-driven atomic structure modeling. First, it should provide a simple interface for users to enter query parameters and seamlessly communicate with the Electron Microscopy Data Bank (EMDB), extract and parse relevant metadata, apply rigorous quality control measures, and retrieve associated structural data—cryoEM maps and atomic models. Second, the tool should generate accurate structural labels and output fully formatted datasets suitable for training and evaluating deep learning models in macromolecular structure prediction. Figure 1 provides an overview of the implemented data processing pipeline of CryoDataBot, which consists of four major modules: Metadata Collection, Metadata Curation, Structural Data Conditioning, and Customized Dataset Construction, along with an illustration of the graphical user interface (GUI).

The **Metadata Collection** module facilitates the gathering of raw data (Fig. 1a). It starts by querying the EMDB using list of user-defined keywords and then automatically collects relevant metadata (Methods). The collected metadata include fields such as entry titles, EMDB IDs, fitted PDB IDs, Q-scores, cross-references to the Universal Protein Knowledgebase (UniProtKB) and AlphaFold, recommended contour levels, and other relevant annotations. To ensure completeness and reduce redundancy, entries missing critical fields (e.g., fitted PDB ID) or containing duplicate EMDB IDs or titles are excluded from the original metadata.

Through the **Metadata Curation** module, the original metadata are subjected to a multi-stage quality control pipeline (Fig. 1b). The first criterion is the Q-score, which quantitatively assesses the atom-level resolvability of cryoEM maps and evaluates the fit between cryoEM maps and their corresponding atomic models. A Q-score of 1.0 represents a perfect fit[46]. Entries with Q-scores below a user-defined threshold are discarded, offering flexible control over map–

model quality. In addition, each entry may include one or more cross-references to UniProtKB and AlphaFold, corresponding to functionally annotated protein domains and their structure predictions, respectively[48,49]. To reduce redundancy, CryoDataBot evaluates whether different entries refer to the same protein domain or structure prediction, and employs a two-tier filtering strategy (Methods), consisting of uniqueness filtering and similarity filtering. This strategy removes highly redundant structures while maintaining dataset diversity. By applying these quality control steps, the curated dataset retains only the most informative, high-confidence entries, enhancing data reliability and reducing unnecessary computational burden during downstream processing.

The workflow of the **Structural Data Conditioning** module is illustrated in Figure 1c. Based on the curated metadata, CryoDataBot automatically retrieves structural data files from EMDB and PDB, obtaining, for each entry, a cryoEM map paired with its corresponding atomic model. To facilitate AI training, the maps, which often have non-uniform voxel sizes, are resampled to user-defined voxel sizes to ensure consistency across the dataset (Methods). After resampling, each map undergoes adaptive density normalization based on its recommended contour level (Methods), ensuring consistent density scaling and providing an inherent denoising effect. This method is more flexible and removes more noise than the fixed-threshold approaches commonly used in prior studies (e.g., DeepTracer[14], Cryo2StructData[22]). To further ensure structural consistency, each map–model pair is under MMF validation using the Volume Overlap Fraction score (VOF score, see Methods), where a high VOF score reflects the accuracy and completeness of the atomic model. Users can apply a configurable threshold to exclude poorly aligned pairs, ensuring that the final dataset consists solely of structurally coherent examples.

Through the **Customized Dataset Construction** module (Fig. 1d), users specify the structures to be labeled, which can include atomic groups (e.g., all atoms within α-helices) or individual atoms (e.g., Cα atoms), along with label values (e.g., 1 or 2). The module generates the corresponding structural labels using the atomic model (Methods). Examples of the resulting labels are shown in the green panel of Fig. 3a and Fig. 4. Once the label data is generated, it is paired with the corresponding cryoEM map and partitioned into smaller 3D sub-volumes based on user-defined stride and patch dimensions. These sub-volumes are then split into training,

validation, and test sets according to user-defined ratios, creating an AI-compatible dataset for downstream learning applications.

Construction of Benchmarking Datasets Using CryoDataBot

State-of-the-art automated modeling tools, such as DeepTracer, ModelAngelo, and CryoREAD, leverage deep learning to build atomic models by identifying protein and RNA secondary structures, localizing key backbone atoms, and classifying residue types[29–31]. To systematically evaluate the effectiveness of CryoDataBot in generating high-quality datasets for structure prediction, we constructed three benchmarking datasets with increasing levels of quality control: a **raw dataset** without quality control, a **control dataset** with basic redundancy filtering, and an **experimental dataset** curated through CryoDataBot's full-quality control pipeline. All three datasets were based on ribosome structures due to their abundance in the EMDB and their inclusion of both protein and RNA components, which are essential for assessing structure prediction across multiple molecule types.

A total of 962 ribosome-related entries were initially retrieved from EMDB using the query "ribosome AND resolution:[3 TO 4]". Following the exclusion of 20 entries due to unsuccessful normalization, 942 entries remained. This unfiltered collection served as the raw dataset, mimicking prior methodologies such as Cryo2StructData[45]. It also provided the foundational pool from which the control and experimental datasets were derived through successive quality control steps. Detailed metadata and a summary of the filtration process are provided in the Supplementary Tables.

Table 2 summarizes the number of entries discarded at each quality control stage for both the control and experimental datasets. For the control dataset, a uniqueness filter was applied to remove 173 fully redundant entries that shared identical UniProtKB cross-references, resulting in a reduced dataset of 769 entries. From this set, 18 entries were randomly selected as a holdout test subset (these entries are not included in the experimental dataset), yielding a final control dataset of 751 entries.

The experimental dataset underwent a more rigorous, multi-stage quality control pipeline (Table 2). First, entries with Q-scores below 0.4 were discarded, removing 406 low-quality entries. In the first tier of redundancy filtering, 92 entries were discarded: 23 due to invalid cross-

references and 69 due to duplication identified through identical UniProtKB annotations. In the second tier, an additional 230 entries exhibiting over 70% similarity were discarded based on UniProtKB-derived similarity evaluation. These quality control steps yielded an experimental dataset of 214 high-confidence, low-redundant entries—approximately one-fourth the size of the raw and control datasets—thus reducing computational overhead while preserving data informativeness. Figure 1b presents representative examples of excluded entries, highlighting improvements in data quality through the removal of structurally incoherent and redundant entries.

Structural data—including cryoEM maps and atomic models—were collected from EMDB and PDB for all three datasets. All cryoEM maps were then resampled to a voxel size of 1 Å, denoised according to their recommended contour levels, and normalized to a 0–1 density scale to ensure uniformity. For the experimental dataset, CryoDataBot further assessed the MMF using the VOF score, excluding an additional 71 entries that fell below the 0.82 threshold (Table 2). This refinement yielded a final set of 143 high-fidelity map–model pairs for the experimental dataset. In comparison, the preprocessed control and raw datasets contained 751 and 942 map–model pairs, respectively.

Using the preprocessed structural data, secondary structure labels were generated for both the control and experimental datasets. The labeled volumes, together with the normalized cryoEM maps, were partitioned into 64^3Å^3 sub-volumes to support batch training. These sub-volumes were subsequently split into training and validation sets at an 80:20 ratio. One entry from the control dataset (EMD-2875) was excluded due to inconsistencies between its cryoEM map and label data, rendering it unsuitable for model training. The resulting control and experimental datasets were thus fully prepared for direct use in U-Net model training workflows.

Quality Assessment of the Constructed Benchmark Datasets

High-quality training datasets are essential for deep learning-based structural modeling. Two critical attributes that determine dataset utility are map–model fitness (MMF) and structural redundancy. Accurate map–model alignment ensures reliable label generation from atomic models, while low redundancy enhances dataset diversity and reduces overfitting risks during model training. To evaluate these attributes, we systematically assessed the raw, control, and experimental datasets curated in this study.

To evaluate MMF, we computed multiple correlation coefficient (CC) metrics for each map–model pair using PHENIX[26], including CC_mask (for atomic center fit), CC_volume (for molecular envelope fit), CC_peaks (for fit of strong peaks), and CC_box (for overall map similarity). These metrics capture different aspects of structural consistency between cryoEM maps and atomic models. As shown in Figure 2a, the experimental dataset consistently outperformed both the raw and control datasets across all CC metrics. Both the raw and control datasets contained a significant number of map–model pairs with CC values below 0.6, indicating poor correspondence between cryoEM maps and atomic models. In contrast, the experimental dataset exhibited fewer low-CC entries and consistently higher 25th, 50th, and 75th percentile values across all CC metrics. These results highlight the effectiveness of Q-score filtering and MMF validation in improving map–model consistency within the dataset, thereby enabling the generation of precise structural labels.

To assess dataset redundancy, we analyzed structural similarity between entries based on InterPro (IPR) domain annotations[50]. IPR identifiers, which represent conserved protein domains with independent folding and functional capabilities, were retrieved for each entry using its PDB ID. Pairwise similarity scores were computed as the ratio of shared IPR identifiers between entry pairs. Figure 2b displays the distribution of similarity scores across all datasets. The experimental dataset shows the highest proportion (64.79%) of least similar pairs (similarity score < 0.2), and the lowest proportion of highly similar pairs (similarity score > 0.6), indicating the lowest overall redundancy. This pattern is further supported by the similarity heatmaps in Figure 2c, where both the raw and control datasets exhibit dense clusters of high-similarity pairs (scores > 0.5), reflecting substantial redundancy from entries with partial or complete structural similarity. In contrast, the experimental dataset shows a more dispersed and localized distribution of high-similarity scores, demonstrating the effectiveness of the redundancy-filtering strategy.

A comparison between the raw and control datasets provides additional insight. The control dataset contains a lower proportion of entry pairs with similarity scores in the 0.8–1.0 range (Fig. 2b), demonstrating the effectiveness of the uniqueness filtering stage in eliminating exact duplicates. However, it has a higher proportion of mid-similarity pairs (0.2–0.6) and a lower proportion of low-similarity pairs (below 0.2). The increase in mid-similarity proportions likely arises from the preservation of partially similar entries, combined with a decrease in the

overall number of entry pairs. Moreover, the excluded redundant entries could have formed low-similarity pairs when matched with structurally unrelated entries; their removal reduces such combinations, contributing to the observed decline in low-similarity proportions. These results suggest that while uniqueness filtering reduces exact redundancy, it is insufficient for addressing broader structural similarities within the dataset.

Performance of U-Net Trained on CryoDataBot-Generated Dataset

To directly assess how dataset quality influences model training and predictive performance, we trained two identical 19-layer 3D U-Net models on the control and experimental datasets, respectively. These models, commonly utilized in AI-based structural modeling frameworks[38], were designed in this study to predict secondary structures from cryoEM maps (Figure 3a). Both U-Net models were trained under identical conditions with consistent learning rates and early stopping criteria. Throughout training and upon completion, model performance was systematically assessed across multiple evaluation dimensions (Methods).

As detailed in Table 3, the experimental dataset substantially outperformed the control dataset in improving training efficiency. Models trained on the experimental dataset achieved faster convergence and higher learning efficiency, requiring significantly fewer epochs before early stopping (130 vs. 162). Figure 3b further corroborates this accelerated convergence, showing a steeper and more consistent decline in loss per epoch for the experimental training (green line), underscoring the dataset's superior quality and informativeness. In addition to faster convergence, the experimental training also demanded significantly less computation, with each epoch completing in just 30 minutes versus 87 minutes for the control—a reduction attributed to the dataset's smaller size. Collectively, these improvements resulted in a more than threefold decrease in total training time (2.7 vs. 9.8 days), substantially accelerating the overall model development process.

At the best-performing epochs (epoch 132 for the control training and epoch 94 for the experimental), the overall accuracy, precision, recall, and F1 score (Methods), all calculated on the validation set, are summarized in Table 3. Despite its substantially smaller size, the experimental dataset enabled the model to outperform the control across all evaluated metrics, achieving higher accuracy (90.14% vs. 88.06%), precision (0.46 vs. 0.41), recall (0.98 vs. 0.96), and F1 score (0.62 vs. 0.57), with consistently better F1 scores across all structural labels. The

superior performance of the model trained on the experimental dataset was consistently evident throughout training (Supplementary Fig. 1).

The previously defined independent test set of 18 cryoEM maps and their corresponding atomic models, randomly selected before training and excluded from both datasets, was used to evaluate both U-Net models. Precision, recall, and F1 scores were independently computed for each structural label within each map (Fig. 3c-e). While the overall F1 scores indicated comparable performance between the experimental and control models, the experimental model consistently demonstrated higher precision across all structural labels, reflecting more accurate and reliable predictions. The recall of the experimental model was slightly lower, which may be explained by the smaller dataset size limiting exposure to structural diversity, or by a more conservative prediction strategy. Nevertheless, the improved precision suggests a favorable trade-off, resulting in a model that is more robust and less prone to generating false positives.

Figure 4 presents two representative test-set examples for the experimental U-Net model: EMD-32074, with the lowest recall, and EMD-3245, with the lowest precision. Blue panels show the predicted secondary structures, restricted to regions with prediction probabilities > 0.8. Green panels display the corresponding atomic models—manually built by the original authors and deposited in the PDB—along with their derived labels, treated as “ground truth”. Evaluation metrics discussed earlier reflect the agreement between these predicted and “ground truth” labels. In the low-recall case (EMD-32074, PDB ID: 7VPX), the deposited atomic model contains substantial inaccuracies, with numerous atoms placed in regions lacking significant cryoEM density. As a result, the “ground truth” labels only partially reflect the actual map features. In contrast, the U-Net model confines its predictions to high-density regions, correctly avoiding unsupported areas, suggesting that the low recall arises from ground truth errors rather than model limitations. Conversely, in the low-precision case (EMD-3245, PDB ID: 3JC2), the atomic model covers only a limited portion of the map, leaving extensive regions of cryoEM density unlabeled. Nevertheless, the U-Net model correctly identifies structural features across the map, such as continuous, well-resolved RNA helices. The resulting low precision thus reflects incomplete “ground truth” labels rather than inaccurate predictions. Together, these examples highlight the model’s robustness against incomplete or noisy ground truth and its

potential utility in refining or validating deposited atomic models by directly leveraging cryoEM maps.

Practical Validation: Retraining CryoREAD with CryoDataBot-Generated Dataset

To further demonstrate the applicability of CryoDataBot-generated datasets within established modeling frameworks, we retrained Stage 1 of CryoREAD—a deep learning framework for de novo modeling of DNA and RNA atomic structures from cryoEM maps[31]. As shown in Figure 5a, Stage 1 is designed to identify and classify key atomic groups—namely sugar, phosphate, base, and base types (A, U/T, C, G)—from the input cryoEM map. In the original CryoREAD study, the Stage 1 training set was constructed through a complex and labor-intensive pipeline involving EMDB map collection, redundancy reduction via clustering, voxel resampling to 1.0 Å, density normalization, and sub-volume extraction, ultimately comprising 290 distinct RNA maps[31]. To replicate this format, we used CryoDataBot to automatically generate new structural labels based on our experimental ribosome dataset consisting of 143 maps, as described in the previous section. The entire process was fully automated by configuring a small set of parameters—including atomic group selection (sugar, phosphate, A, U/T, C, G), resampling voxel size (1.0 Å), labeling radius (2.0 Å), and sub-volume size (64^3 Å^3)—without requiring further manual intervention. Training was performed using the same hyperparameter settings as in the original CryoREAD training. Evaluation was conducted on a consistent test set comprising 63 entries, obtained by removing five overlapping or invalid cases (EMD-10535, EMD-6789, EMD-21856, EMD-9572, and EMD-4138) from the original 68.

Evaluation results for the retrained Stage 1 model are shown in Figures 5b-d (green), alongside those of the original model (yellow). For structure detection, the median F1 scores (Fig. 5b) for sugar, phosphate, and base are 0.619, 0.540, and 0.760, respectively, reflecting the model's ability to accurately localize fundamental components of nucleic acid structures. For fine-grained base classification, the median F1 scores for individual base types—A, U/T, C, and G—are 0.473, 0.406, 0.467, and 0.541, respectively. These results indicate that the retrained model achieves approximately 50% accuracy for each base type, substantially exceeding the 25% expected by random chance among four classes. These F1 scores are comparable to those of the original model, demonstrating that CryoREAD, when trained on CryoDataBot-generated datasets, retains high predictive performance. This is particularly notable given that the

CryoDataBot-generated training set was created without labor-intensive manual curation, is smaller in size, and is limited to ribosome-derived biomacromolecules, in contrast to the broader range of RNA structures included in the original CryoREAD training set.

The precision (Fig. 5c) and recall (Fig. 5d) for structure detection again reveal a trade-off. Compared to the original training, training on CryoDataBot-generated dataset results in higher precision but lower recall for detecting sugar (precision: 0.679 vs. 0.556; recall: 0.644 vs. 0.719) and base (precision: 0.781 vs. 0.744; recall: 0.787 vs. 0.825). However, phosphate detection shows the opposite trend, with lower precision (0.512 vs. 0.623) but higher recall (0.647 vs. 0.568) when trained on CryoDataBot-generated dataset. The observed changes in precision and recall are non-negligible, underscoring that training set composition can differentially affect model performance. This highlights the importance of selecting training data carefully according to study-specific goals, with attention to factors such as structural diversity, data quality, and preprocessing settings.

Figure 6 illustrates a representative example (EMD-3532) of RNA atomic group prediction by the retrained CryoREAD Stage 1 model. As in previous evaluations, the ground truth was derived from the deposited atomic model, manually built by the original authors and available in the PDB. The green panel shows the cryoEM map (gray) overlaid with the ground truth model (spheres). The blue panels (Fig. 6b–g) present Stage 1 predictions, rendered as transparent volumes with probability > 0.4, overlaid with the ground truth atomic model in ball-and-stick format to facilitate direct structural comparison. The composite prediction (Fig. 6a) omits regions corresponding to protein density—as indicated by the white cartoon representation of the protein model in Fig. 6h—and accurately reconstructs the canonical RNA double helix. The sugar (pink) and phosphate (red) groups form the backbone, while the four types of bases are embedded between the helices. The predicted structure closely aligns with the ground truth, not only in the overall RNA topology but also in the precise localization of atomic groups and accurate classification of base types (Fig. 6b-g). This high level of accuracy provides a strong foundation for downstream automated modeling stages in CryoREAD. Overall, the example demonstrates the effectiveness of deep learning in interpreting complex cryoEM maps and underscores the robustness of the CryoDataBot-generated training data in enabling reliable structure prediction.

Discussion

To meet the critical need for high-quality cryoEM datasets in AI-driven structural modeling, we developed CryoDataBot, which, to our knowledge, is the first software specifically designed to streamline and scale the generation of standardized, high-quality datasets essential for robust model development. Many widely adopted AI frameworks still rely on datasets that are either unfiltered or lack systematic quality control, introducing biases and limiting the ability to fairly evaluate and compare model architectures across tools. CryoDataBot addresses this challenge by automating the data construction pipeline while enforcing rigorous, standardized quality control. This not only improves the reliability of downstream AI models but also enables fair benchmarking based on a consistent dataset foundation. Furthermore, CryoDataBot offers extensive flexibility, allowing users to configure nearly all processing parameters with fine granularity based on specific modeling objectives and available data, thereby accommodating the diverse needs of structural biology tasks.

We demonstrate that AI models trained on datasets generated by CryoDataBot achieve notably higher precision, reflecting more accurate and reliable predictions. However, models trained on strictly filtered datasets sometimes show a modest decrease in recall. This trade-off likely results from the reduced dataset size caused by rigorous filtering, which enhances accuracy but limits exposure to structural variability. Researchers are therefore encouraged to adjust the quality control stringency according to their scientific objectives. For applications requiring high-confidence predictions—such as identifying accurate protein sequences from unknown cryoEM maps—a rigorously filtered dataset is preferable. In contrast, when the goal is to capture broader structural features, such as in automated de novo modeling where further refinement is expected, a more relaxed threshold may improve recall by preserving diversity.

As AI continues to transform cryoEM-based structural biology, the ability to automatically generate high-quality, customizable datasets is becoming essential for next-generation AI tool development. CryoDataBot fulfills this need as a rare, purpose-built solution that reduces the technical burden of dataset construction and facilitates broader integration of AI into structural biology research.

The introduction of CryoDataBot marks a significant advancement in cryoEM data handling, offering a robust tool that standardizes dataset creation and supports the growing intersection of structural and computational biology. By ensuring high-quality, curated data, CryoDataBot helps overcome one of the key limitations in AI-based cryoEM modeling: dependence on unreliable or incomplete data. This work not only streamlines dataset preparation but also lays the foundation for more precise and reproducible model predictions. As AI tools evolve, CryoDataBot's flexibility will be critical for adapting to emerging modeling approaches, supporting collaboration across structural biology, computational biology, and related fields.

The impact of this work extends beyond model accuracy. With standardized, customizable datasets, CryoDataBot enables transparent benchmarking and cross-validation of AI models. Such transparency may help establish best practices in AI-driven structural biology, fostering a collaborative environment where models can be fairly compared on a consistent data foundation. Moreover, CryoDataBot's adaptability ensures its continued relevance as cryoEM and AI technologies advance, positioning it as a vital tool in the field for years to come.

Methods

Retrieving Metadata for EMDB Entries

Our tool searches the EMDB for relevant entries based on user-defined keywords, such as molecule type, organism, and resolution range. In the Electron Microscopy Data Bank (EMDB), each deposited cryoEM map is assigned a unique EMDB ID (e.g., "EMD-1234"). These cryoEM maps are often associated with corresponding atomic models, which have been fitted to the cryoEM maps. The atomic models are deposited separately in the Protein Data Bank (PDB) and are identified by their own PDB IDs (e.g., "6XYZ" or "7ABC"). CryoDataBot automatically retrieves a list of EMDB IDs and their associated fitted PDB IDs along with other metadata. Key metadata fields include: EMDB ID, fitted PDB IDs, the entry title, resolution (in Ångströms), UniProtKB cross-references, AlphaFold cross-references, Q-score (a quantitative measure assessing atom resolvability in cryoEM maps), atom inclusion (the percentage of fitted atoms present in the density map), and the recommended contour level (the optimal threshold for visualizing electron density in 3D reconstructions).

Two-Tier Redundancy Filtering

Certain entries may represent the same biomacromolecule, though they are not exact duplicates. A biomacromolecule may have multiple EMDB entries due to variations in its conformational states or its binding to different ligands. To mitigate the overrepresentation of identical biomacromolecules or redundant structures within similar biomacromolecules, CryoDataBot employs a two-tier redundancy filtering process.

To assess the similarity between entry pairs, CryoDataBot leverages UniProtKB and AlphaFold cross-references, which correspond to functionally annotated protein domains and their respective structural predictions. Structural redundancy between pairs is quantified based on the proportion of shared cross-references, with a greater overlap signifying a higher degree of shared protein domains and, consequently, greater structural similarity.

The first-tier filtering, uniqueness filtering, removes entries with entirely overlapping cross-references, ensuring that only the highest-resolution entry is retained for each redundant group. Furthermore, uniqueness filtering flags and removes entries lacking both UniProtKB and AlphaFold cross-references. These flagged entries are stored separately for manual review, with the option to re-add them if deemed useful.

The second-tier filtering, similarity filtering, offers users the option to customize the desired level of dataset redundancy. Similarity filtering discards entries where the overlap ratio of cross-references exceeds a user-defined threshold, while retaining the entry with the highest resolution within each redundant group. This approach provides flexible control over dataset redundancy, allowing users to tailor the redundancy level to their specific needs, as the degree of redundancy directly influences the dataset size.

Customized Map Resampling and Adaptive Map Normalization

The methodology in this study provides a more efficient and flexible approach to cryoEM map conditioning. While previous methods (e.g., DeepTracer[29], Cryo2StructData[45]) rely on ChimeraX[28] for map resampling, CryoDataBot leverages the *cupyx.scipy.ndimage.zoom* function from the CuPy Python library. This tool, integrated into the overall Python code, enables the resampling of cryoEM maps into a customizable uniform voxel size, with a default configuration of 1.0 Å × 1.0 Å × 1.0 Å. It eliminates the dependency on ChimeraX, thereby improving both the efficiency and flexibility of the resampling process.

In addition, our normalization approach for map densities addresses key limitations found in previous methods. These methods apply a fixed denoising threshold of zero, eliminating density values below zero for each map, retaining positive values, and normalizing the resulting density values to the 0–1 range[29,31,45]. While effective to some degree, the previous approach does not account for variability in value ranges and noise levels across different cryoEM density maps, potentially leading to suboptimal denoising and normalization.

To overcome these challenges, we introduce adaptive thresholds tailored to each density map. These thresholds are calculated based on the recommended contour level specified in the map's metadata, ensuring that the recommended contour level corresponds to the 85th percentile of density values above the threshold. Density values below this threshold are eliminated, while those above it are retained and normalized to the 0–1 range. By adapting the threshold to the unique characteristics of each map, our approach enhances the precision of the denoising process and improves the adaptability of normalization, leading to more accurate and reliable results.

Map–model Fitness (MMF) Validation

While Q-score provides a useful residue-level assessment of model quality, it is insufficient for detecting global inconsistencies between cryoEM maps and their corresponding atomic models. In particular, it often fails to filter out entries with localized agreement but poor overall alignment—such as cases where large regions of the cryoEM map remain unmodeled, or substantial portions of the model are unsupported by density (see the MMF validation example in Fig. 1c). To address these limitations, we implemented an additional validation step, termed MMF validation, following map and model download, to more thoroughly evaluate global structural consistency.

To quantify global map–model alignment, we compute a Volume Overlap Fraction (VOF) score as the primary fitness metric. After map normalization, both the cryoEM map and the atomic model are projected into 2D along six directions—the three principal axes (X, Y, Z) and three diagonal-like orientations. Each projection is obtained by summing voxel values along the axis orthogonal to the projection plane (e.g., summing along the Z-axis for the XY projection). The resulting 2D images are then binarized by setting all values ≥ 1 to 1 and values < 1 to 0. For each projection pair, we calculate the intersection-over-union (IoU), or Jaccard index[51], defined as the ratio of overlapping pixels to the total number of unique pixels in both

projections. The final VOF score is computed as the average IoU across all six projections, excluding the highest score to reduce directional bias. Entries with VOF scores below a user-defined threshold (ranging from 0 to 1, with 1 indicating perfect alignment) are discarded.

As a secondary metric, we also compute a Dice-like coefficient for each projection, defined as the pixel overlap divided by the total number of pixels across both projections (i.e., a Dice coefficient without the factor of two). The average Dice-like score is reported alongside the VOF score to provide complementary insight into map–model consistency.

Generating Structural Labels

Structural labels are generated from atomic models. A 3D voxel array is initialized to match the spatial dimensions of the corresponding resampled cryoEM map and is then superimposed with the atomic model. Users specify label values (e.g., 1, 2, 3, 4) for atomic groups of interest, such as ligands, secondary structures, amino acids, or individual atoms.

The atomic model is systematically traversed chain by chain to extract the coordinates of the specified atomic groups. These coordinates, representing atom positions, are transformed into voxel indices within the superimposed empty array. Label values are then assigned to voxels within a user-defined Euclidean distance of the target indices. If a voxel falls within the labeling radius of multiple atomic groups, the algorithm assigns the label of the nearest one.

Training the U-Net model

As shown in Fig. 3a, we trained two independent 19-layer 3D U-Net models to predict secondary structures from cryoEM maps. Each model takes a 64^3 Å^3 density sub-volume as input and outputs N=5 probability volumes of the same size, representing voxel-wise probabilities for 5 classes: *Nothing*, *Helix*, *Sheet*, *Coil*, and *RNA*. Both models were trained under identical settings using a batch size of 16, the Adam optimizer[52] with a learning rate of 1×10^{-4}, and a weighted cross entropy loss to address class imbalance, with class weights set to [1, 57, 128, 52, 22]. Training was carried out on an NVIDIA GeForce RTX 4090 GPU with 24 GB of display memory. We applied early stopping with a patience of 30 epochs, leading to training durations of 9.8 days (162 epochs) and 2.7 days (130 epochs) on the control and experimental datasets, respectively. The best-performing checkpoints were selected at epoch 132 for the control model

and epoch 94 for the experimental model. Training and validation loss curves are provided in Figure 3b.

Evaluating AI model prediction performance

The outputs of both the U-Net model and CryoREAD Stage 1 are voxel-based, with each voxel assigned a probability distribution over all structural labels. For example, in the U-Net model predictions, each voxel contains a probability vector (p_0, p_1, p_2, p_3, p_4), representing the probabilities of the voxel belonging to one of five classes: 0, 1, 2, 3, 4, corresponding to *Nothing*, *Helix*, *Sheet*, *Coil*, and *RNA*, respectively. For evaluation, a voxel is assigned to label k ($k \in \{1, 2, 3, 4\}$) if p_k exceeds 0.8 (or 0.4 for CryoREAD Stage 1, as recommended by its first author); when none of the probabilities exceed the threshold, the voxel is assigned to label 0 (*Nothing*). Ground truth labels were generated from deposited atomic models using the same procedure described in the **Generating Structural Labels** section.

To evaluate prediction performance for each label, we computed voxel-wise accuracy, precision, recall, and F1 score. For a given label *k*, TP, FP, FN, and TN denote the number of voxels correctly predicted as *k*, incorrectly predicted as *k*, missed ground truth voxels of *k*, and correctly predicted as not *k*, respectively. The evaluation metrics are calculated as follows:

$$\textbf{Accuracy} = (TP + TN) / (TP + TN + FP + FN)$$

$$\textbf{Precision} = TP / (TP + FP)$$

$$\textbf{Recall} = TP / (TP + FN)$$

$$\textbf{F1 Score} = 2 \times (Precision \times Recall) / (Precision + Recall)$$

Accuracy measures the proportion of all predicted labels, across all classes, that match the ground truth. Precision quantifies the fraction of correct predictions among all predicted labels, reflecting the reliability of the model's outputs. Recall captures the proportion of ground truth labels that were correctly identified, indicating the model's sensitivity to relevant features. The F1 score, defined as the harmonic mean of precision and recall, provides a balanced evaluation of both correctness and coverage—particularly useful when class distributions are imbalanced or when precision and recall diverge. These metrics were computed separately for

each structural label and in aggregate, both during and after training, to enable detailed performance assessment.

Graphical User Interface (GUI) for the Tool

To enhance usability, we developed a modern and user-friendly GUI using PyQt5, a Python binding for the cross-platform Qt framework. The interface (Fig. 1e) features an intuitive design that streamlines data retrieval, quality control, and label selection, thereby facilitating efficient user interaction with the tool.

For data retrieval, users can formulate queries using EMDB's native search syntax[47]. The application then fetches and downloads a comma-separated values (CSV) file containing the corresponding entries and associated metadata. For quality control, users may proceed with the retrieved CSV file or upload a custom CSV file, followed by the specification of filtering parameters to curate the dataset. For label selection, the interface allows users to define atomic groups by configuring options across three hierarchical categories: the **secondary structure** category includes *helix*, *sheet*, *coil*, and *RNA*; the **residue type** category supports the 20 standard amino acids and the 4 canonical nucleobases; and the **atom type** category allows input of specific atom names in PDB files (e.g., *CA*, *Mg*). For instance, to label all Cα atoms within helices, the user would select "helix" under secondary structure, leave residue type unspecified, and input "CA" as the atom type.

Data Availability

The entries of the cryoEM maps and their corresponding atomic models used in this study are listed in the Supplementary Tables. The cryoEM maps can be downloaded from the EMDB via the European Molecular Biology Laboratory – European Bioinformatics Institute (EMBL-EBI) FTP server (https://ftp.ebi.ac.uk/pub/databases/emdb/structures). The corresponding atomic models are available from the Research Collaboratory for Structural Bioinformatics (RCSB, https://www.rcsb.org). All data that support this study are available from the corresponding authors upon request.

Code Availability

The source code of CryoDataBot is available at https://github.com/t00shadow/CryoDataBot under the MIT license.

References

1. Petsko, G. A. & Ringe, D. Protein Structure and Function. (New Science Press, 2004).
2. Branden, C. I. & Tooze, J. Introduction to Protein Structure. (Garland Science, 2012).
3. Zhou, K. et al. Atomic model of vesicular stomatitis virus and mechanism of assembly. Nat. Commun. 13, 5980 (2022).
4. Liu, S., Xia, X., Calvo, E. & Zhou, Z. H. Native structure of mosquito salivary protein uncovers domains relevant to pathogen transmission. Nat. Commun. 14, 899 (2023).
5. Liu, X., Xia, X., Martynowycz, M. W., Gonen, T. & Zhou, Z. H. Molecular sociology of virus-induced cellular condensates supporting reovirus assembly and replication. Nat. Commun. 15, 1–12 (2024).
6. Liu, S., Su, T., Xia, X. & Zhou, Z. H. Native DGC structure rationalizes muscular dystrophy-causing mutations. Nature 637, 1261–1271 (2025).
7. Zheng, H. et al. X-ray crystallography over the past decade for novel drug discovery – where are we heading next? Expert Opin. Drug Discov. 10, 975–989 (2015).
8. Hu, Y. et al. NMR-Based Methods for Protein Analysis. Anal. Chem. 93, 1866–1879 (2021).
9. Cai, X. et al. Identification and architecture of a putative secretion tube across mycobacterial outer envelope. Sci. Adv. 7, eabg5656 (2021).
10. Wang, Y. et al. Structure of LARP7 protein p65–telomerase RNA complex in telomerase revealed by cryo-EM and NMR. J. Mol. Biol. 435, 168044 (2023).
11. Cheng, Y. Single-particle cryo-EM at crystallographic resolution. Cell 161, 450–457 (2015).
12. Bai, X.-C., McMullan, G. & Scheres, S. H. How cryo-EM is revolutionizing structural biology. Trends Biochem. Sci. 40, 49–57 (2015).
13. Zhu, K.-F. et al. Applications and prospects of cryo-EM in drug discovery. Mil. Med. Res. 10, 10 (2023).
14. Saibil, H. R. Cryo-EM in molecular and cellular biology. Mol. Cell 82, 274–284 (2022).
15. Ho, C.-M. et al. Bottom-up structural proteomics: cryoEM of protein complexes enriched from the cellular milieu. Nat. Methods 17, 79–85 (2020).

16. Agnew, A., Humm, E., Zhou, K., Gunsalus, R. P. & Zhou, Z. H. Structure and identification of the native PLP synthase complex from Methanosarcina acetivorans lysate. mBio 16, e03090-24 (2025).

17. Ho, C.-M. et al. Native structure of the RhopH complex, a key determinant of malaria parasite nutrient acquisition. Proc. Natl. Acad. Sci. 118, e2100514118 (2021).

18. Wang, H. et al. Composition and in situ structure of the Methanospirillum hungatei cell envelope and surface layer. Sci. Adv. 10, eadr8596 (2024).

19. Li, X. et al. Electron counting and beam-induced motion correction enable near-atomic-resolution single-particle cryo-EM. Nat. Methods 10, 584–590 (2013).

20. McMullan, G., Chen, S., Henderson, R. & Faruqi, A. R. Detective quantum efficiency of electron area detectors in electron microscopy. Ultramicroscopy 109, 1126–1143 (2009).

21. Rohou, A. & Grigorieff, N. CTFFIND4: Fast and accurate defocus estimation from electron micrographs. J. Struct. Biol. 192, 216–221 (2015).

22. Grant, T., Rohou, A. & Grigorieff, N. cis TEM, user-friendly software for single-particle image processing. elife 7, e35383 (2018).

23. Moriya, T. et al. High-resolution single particle analysis from electron cryo-microscopy images using SPHIRE. J. Vis. Exp. JoVE 55448 (2017).

24. Tang, G. et al. EMAN2: an extensible image processing suite for electron microscopy. J. Struct. Biol. 157, 38–46 (2007).

25. Gao, Y., Thorn, V. & Thorn, A. Errors in structural biology are not the exception. Biol. Crystallogr. 79, 206–211 (2023).

26. Adams, P. D. et al. PHENIX: a comprehensive Python-based system for macromolecular structure solution. Biol. Crystallogr. 66, 213–221 (2010).

27. Emsley, P. & Cowtan, K. Coot: model-building tools for molecular graphics. Biol. Crystallogr. 60, 2126–2132 (2004).

28. Meng, E. C. et al. UCSF ChimeraX: Tools for structure building and analysis. Protein Sci. 32, e4792 (2023).

29. Pfab, J., Phan, N. M. & Si, D. DeepTracer for fast de novo cryo-EM protein structure modeling and special studies on CoV-related complexes. Proc. Natl. Acad. Sci. 118, e2017525118 (2021).

30. Jamali, K., Kimanius, D. & Scheres, S. ModelAngelo: Automated Model Building in Cryo-EM Maps. ArXiv Prepr. ArXiv221000006 (2022).
31. Wang, X., Terashi, G. & Kihara, D. CryoREAD: de novo structure modeling for nucleic acids in cryo-EM maps using deep learning. Nat. Methods 1–9 (2023) doi:10.1038/s41592-023-02032-5.
32. Liu, Y.-T., Fan, H., Hu, J. J. & Zhou, Z. H. Overcoming the preferred-orientation problem in cryo-EM with self-supervised deep learning. Nat. Methods 22, 113–123 (2025).
33. Jih, J., Liu, Y.-T., Liu, W. & Zhou, Z. H. The incredible bulk: Human cytomegalovirus tegument architectures uncovered by AI-empowered cryo-EM. Sci. Adv. 10, eadj1640 (2024).
34. Liu, Y.-T. et al. Isotropic reconstruction for electron tomography with deep learning. Nat. Commun. 13, 1–17 (2022).
35. Kinman, L. F., Carreira, M. V., Powell, B. M. & Davis, J. H. Automated model-free analysis of cryo-EM volume ensembles with SIREn. Structure 33, 974–987 (2025).
36. Zhang, H. et al. CryoPROS: Correcting misalignment caused by preferred orientation using AI-generated auxiliary particles. Nat. Commun. 16, 1–16 (2025).
37. Xu, K., Wang, Z., Shi, J., Li, H. & Zhang, Q. C. A2-net: Molecular structure estimation from cryo-em density volumes. in Proceedings of the AAAI Conference on Artificial Intelligence vol. 33 1230–1237 (2019).
38. Farheen, F., Terashi, G., Zhu, H. & Kihara, D. AI-based methods for biomolecular structure modeling for Cryo-EM. Curr. Opin. Struct. Biol. 90, 102989 (2025).
39. Zhang, X., Zhang, B., Freddolino, L. & Zhang, Y. CR-I-TASSER: assemble protein structures from cryo-EM density maps using deep convolutional neural networks. Nat. Methods 19, 195–204 (2022).
40. Terashi, G., Wang, X., Prasad, D., Nakamura, T. & Kihara, D. DeepMainmast: integrated protocol of protein structure modeling for cryo-EM with deep learning and structure prediction. Nat. Methods 21, 122–131 (2024).
41. Zhao, Y., Chen, J. & Oymak, S. On the Role of Dataset Quality and Heterogeneity in Model Confidence. Preprint at https://doi.org/10.48550/arXiv.2002.09831 (2020).
42. Mohammed, S. et al. The Effects of Data Quality on Machine Learning Performance. Preprint at https://doi.org/10.48550/arXiv.2207.14529 (2024).
43. EMDB - the electron microscopy data bank. Nucleic Acids Res. 52, D456–D465 (2024).

44. Burley, S. K. et al. Protein Data Bank (PDB): The Single Global Macromolecular Structure Archive. in Protein Crystallography (eds. Wlodawer, A., Dauter, Z. & Jaskolski, M.) vol. 1607 627–641 (Springer New York, New York, NY, 2017).

45. Giri, N., Wang, L. & Cheng, J. Cryo2structdata: A large labeled cryo-em density map dataset for ai-based modeling of protein structures. Sci. Data 11, 458 (2024).

46. Pintilie, G. et al. Measurement of atom resolvability in cryo-EM maps with Q-scores. Nat. Methods 17, 328–334 (2020).

47. Duraisamy, A. K., Fonseca, N., Kleywegt, G. J., Patwardhan, A. & Morris, K. L. EMICSS: Added-value annotations for EMDB entries. Preprint at https://doi.org/10.48550/arXiv.2501.10882 (2025).

48. UniProt: the Universal protein knowledgebase in 2025. Nucleic Acids Res. 53, D609–D617 (2025).

49. Varadi, M. et al. AlphaFold Protein Structure Database: massively expanding the structural coverage of protein-sequence space with high-accuracy models. Nucleic Acids Res. 50, D439–D444 (2022).

50. Paysan-Lafosse, T. et al. InterPro in 2022. Nucleic Acids Res. 51, D418–D427 (2023).

51. Jaccard, P. Étude comparative de la distribution florale dans une portion des Alpes et des Jura. Bull Soc Vaudoise Sci Nat 37, 547–579 (1901).

52. Kingma, D. P. & Ba, J. Adam: A Method for Stochastic Optimization. Preprint at https://doi.org/10.48550/arXiv.1412.6980 (2017).

Contributions

Z. Hong Zhou conceived and oversaw the project, participated in project design and result illustration, and wrote the paper. Qibo Xu co-conceived the project and participated in all aspects of its development. Leon Wu designed and built the GUI, assisted with debugging, optimizing, and organizing the backend code, prepared Figures 1e and 3b, and drafted the Methods section. Michael Rebelo developed the redundancy evaluation method, contributed to backend debugging and optimization, and assisted with manuscript writing. Shi Feng implemented the Linux command-line interface, optimized the customized dataset construction module, contributed to the metadata collection and structural data conditioning modules as well as the redundancy evaluation method, prepared Figures 2b and 2c, and drafted the Methods section. Xinye Yu developed the Q-score fetching function, helped test and optimize the software, assisted in creating Figures 4 and 6, and drafted the Discussion section. Farhanaz Farheen retrained CryoREAD, evaluated the results, helped prepare Figure 5, and drafted the first paragraph of the CryoREAD retraining section. Daisuke Kihara supervised the CryoREAD retraining and evaluated the results. All authors edited and approved the manuscript.

Competing Interests

The authors declare no competing interest.

Acknowledgements

This project is supported by a grant from the US National Institutes of Health (R01GM071940 to Z.H.Z.). D.K. acknowledges supports from the NIH (R01GM133840) and the National Science Foundation (IIS2211598).

We thank Xiao Wang for technical help regarding training and evaluation of the retrained CryoREAD model.

Software		DeepTracer	ModelAngelo	CryoREAD	Cryo2StructData	**CryoDataBot**
**Dataset Overview**	**Macromolecule Type**	protein	protein, RNA & DNA	RNA & DNA	protein	**Customized***
	**Entries (Initial)**	1,800	3,715	1,384	7,600	**Customized**
	**Entries (Curated)**	1,800	~ 700	290	7,600	**Customized**
	**Resample Voxel Size**	0.5 Å	1.5 Å	1.0 Å	1.0 Å	**Customized**
**Quality Enhancement Procedures**	**MMF Evaluation**	No	**Yes**	**Yes**	No	**Yes**
	**Redundancy Handling**	No	**Yes****	**Yes**	No	**Yes**
**Public Availability**		No	No	No	**Yes**	**Yes**

* user-defined; can include proteins, DNA, RNA, or specific macromolecular types such as ribosomes, spliceosomes, etc.

** manual work involved

Table 1: Comparison of training datasets used by existing AI-driven cryoEM modeling tools, Cryo2StructData dataset, and CryoDataBot-generated dataset. Notably, while some tools have implemented quality enhancement procedures, their datasets exhibit non-uniform voxel sizes and remain unavailable to the public. Cryo2StructData offers a large, accessible dataset, but omits several key quality control steps. Furthermore, existing datasets lack flexibility, limiting researchers' ability to tailor data for specific biomolecular targets or analytical needs. CryoDataBot addresses these limitations by enabling fully customizable, quality-controlled dataset generation.

Entry Count (Pre-QC)	942		
**Quality Control Stages**	**Raw dataset**	**Control dataset**	**Experimental dataset**
Q-score Filter (Threshold: 0.4)	✗	✗	✓ (-406)
Uniqueness Filtering	✗	✓ (-173)	✓ (-92)
Similarity Filtering (Threshold: 70%)	✗	✗	✓ (-230)
MMF Validation (Threshold: 0.82)	✗	✗	✓ (-71)
Entry Count (Post-QC)	942	769	143

Table 2: Quality control (QC) stages applied to construct the raw, control and experimental datasets. Entry counts are provided before and after QC stages, with values in parentheses indicating the number of entries discarded during each stage. After undergoing the full QC pipeline, the final experimental dataset comprises only 143 entries. This was achieved by eliminating highly redundant and structurally incompatible entries, thereby yielding a more compact, reliable dataset for training.

Training set	Control dataset	Experimental dataset
Early stopping epochs	162	130
Training time per epoch (min)	87	30
Total training time (day)	9.8	2.7
Accuracy	88.06%	90.14%
Precision	0.41	0.46
Recall	0.96	0.98
F1 score	0.57	0.62
F1 score for each label	(0.51, 0.54, 0.47, 0.63)	(0.58, 0.60, 0.57, 0.66)

Table 3. Comparison of U-Net model training efficiency and post-training performance on the control and experimental datasets. Early stopping epochs, training time per epoch, and total training time were systematically collected to evaluate training efficiency. Post-training evaluation was performed on the validation set using the best-performing epochs (epoch 132 for the control dataset and epoch 94 for the experimental). Key performance metrics, including overall accuracy, precision, recall, and F1 score, were calculated to assess model prediction performance. F1 scores for helix, sheet, coil, and RNA predictions are shown in parentheses. Across all evaluation criteria, the experimental dataset consistently demonstrated superior performance over the control dataset.

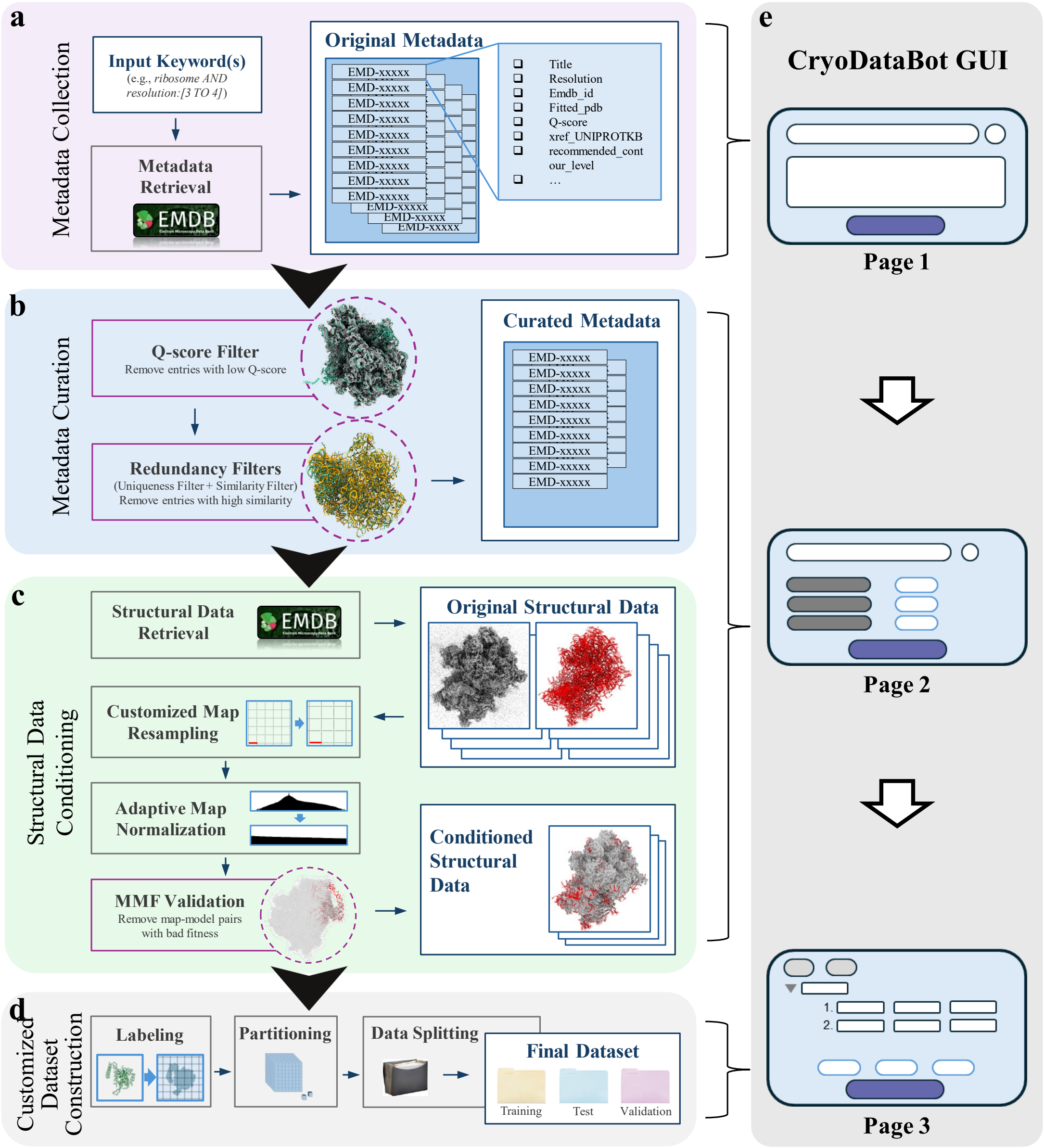

Fig. 1: Pipeline of CryoDataBot. CryoDataBot consists of four functional modules. **a**, Metadata Collection: entries are retrieved from EMDB using user-defined keywords. **b**, Metadata Curation: metadata undergo a multi-stage quality control pipeline to ensure map–model fitness and reduce overall redundancy. **c**, Structural Data Conditioning: cryoEM maps and atomic models are retrieved, resampled to a customized uniform voxel size, and normalized to 0–1 density range using recommended contour levels for denoising. Map–model fitness is then validated to ensure the accuracy and completeness of the atomic models. **d**, Customized Dataset Construction: conditioned structural data are labeled based on user-specified atom types, partitioned into 3D sub-volumes, and split into training, validation, and test sets tailored for AI applications. For further details, see Methods. **e**, Cartoon representation of the graphical user interface (GUI) of CryoDataBot. The first page of the GUI handles module **a**, the second page covers modules **b** and **c**, and the third page is dedicated to module **d**.

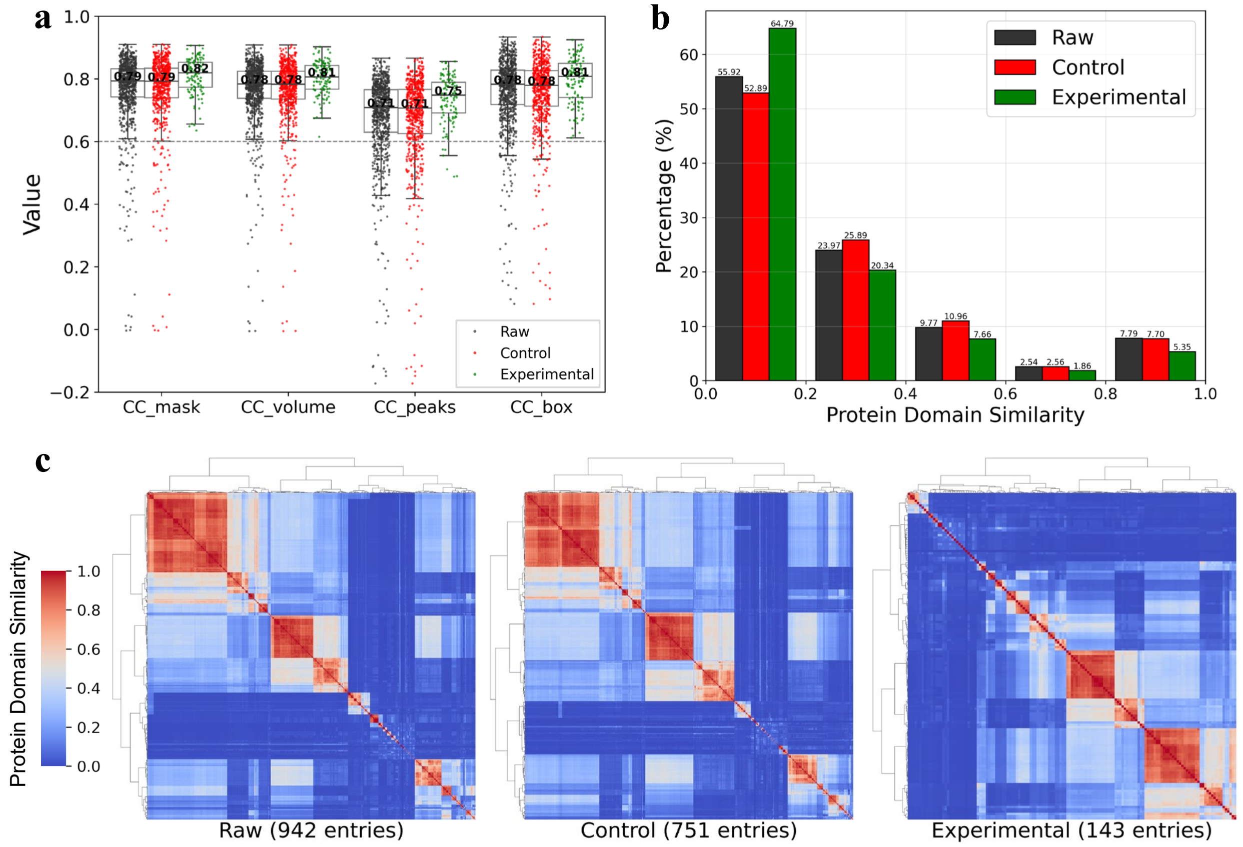

Fig. 2: Evaluation of map–model fitness and structural redundancy in raw, control, and experimental datasets. a, Correlation coefficient (CC) metrics showing that the experimental dataset (green) consistently outperformed both the raw (dark gray) and control (red) datasets. The statistics were calculated over n = 942, 751, and 143 independent cryoEM maps in the raw, control, and experimental datasets, respectively. The numbers in the middle of the box plots represent the median values for each CC metric across the datasets. The gray dashed line in the figure represents a CC value of 0.6. **b**, A histogram illustrating the percentage distribution of pairwise protein domain similarity scores across the three datasets. The x-axis indicates similarity score ranges, with the values above each bar denoting the proportion of each dataset within those ranges. The experimental dataset shows the highest proportion of least similar pairs (below 0.2) and the lowest proportions of mid- (0.2-0.6) and high-similarity pairs (above 0.8), indicating reduced redundancy. **c,** Clustered heatmaps of pairwise protein domain similarity matrix across the three datasets, with warmer colors indicating higher similarity. The raw and control datasets show dense clusters of high-similarity pairs (values > 0.5), while the experimental dataset shows a more dispersed and localized distribution of high-similarity pairs.

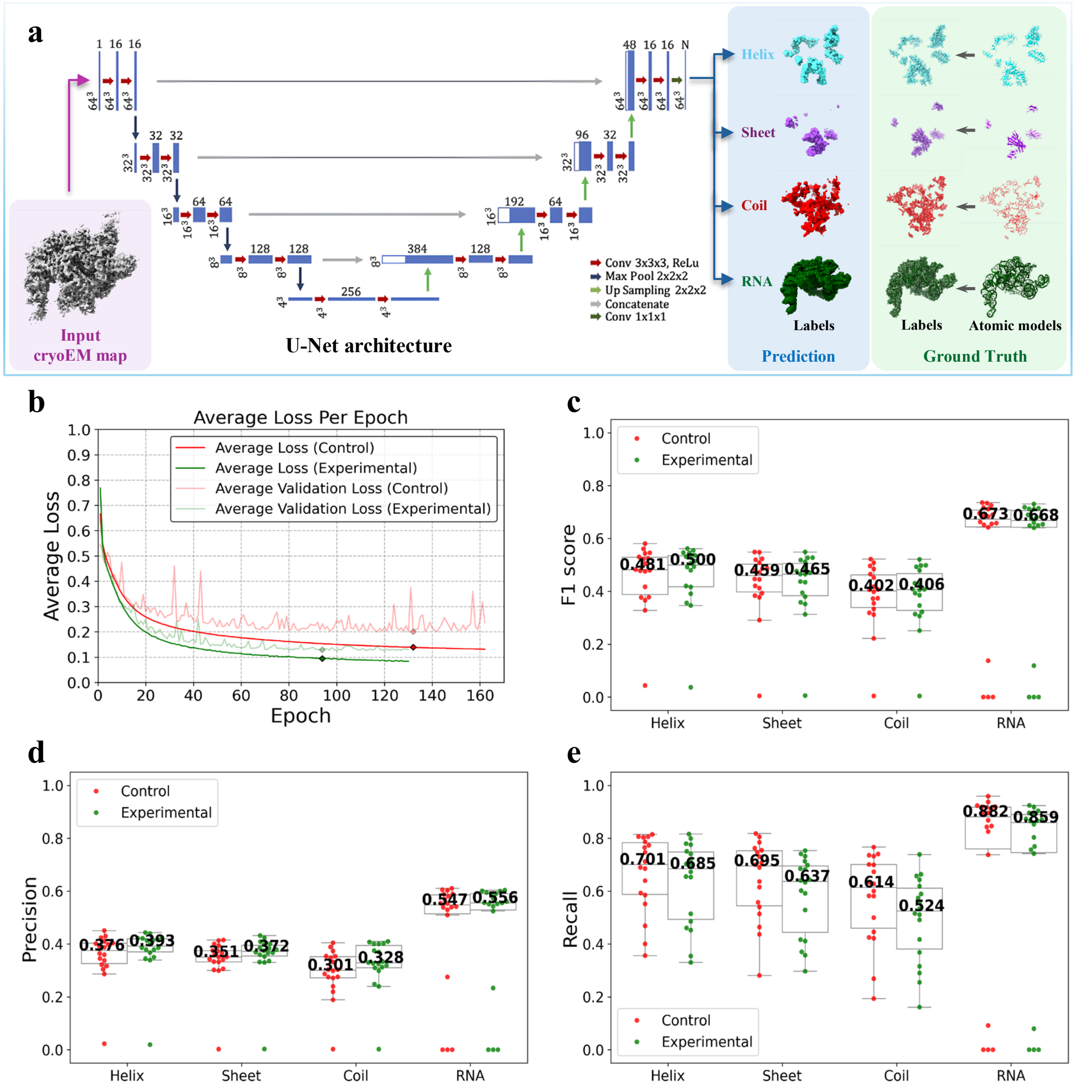

Fig. 3: Evaluation of dataset performance in U-Net model training. a, Schematic of a 19-layer 3D U-Net architecture for secondary structure prediction from cryo-EM maps. The input (purple panel) is the cryoEM map, and the the output (blue panel) contains predicted labels for helices, sheets, coils, and RNA. Ground truth labels derived from atomic models are shown in the green panel. The central diagram illustrates the U-Net architecture, adapted from DeepTracer. Blue bars represent feature maps, with spatial dimensions indicated at the bottom left and the number of channels at the top. **b**, Average loss curves for control (red) and experimental (green) training. Solid lines represent training loss, and lighter lines represent validation loss. The control and experimental models were trained for 162 and 130 epochs, respectively, with the best-performing epochs marked at 132 and 94 (diamonds). **c**, F1 scores for each structural label, calculated on an independent test set of n = 18 cryoEM maps using the U-Net model trained on the control dataset (red) and the experimental dataset (green) at their respective best-performing epochs. The numbers at the middle of the box plots indicate the median values. **d-e**, Same as **c**, but for precision and recall, respectively.

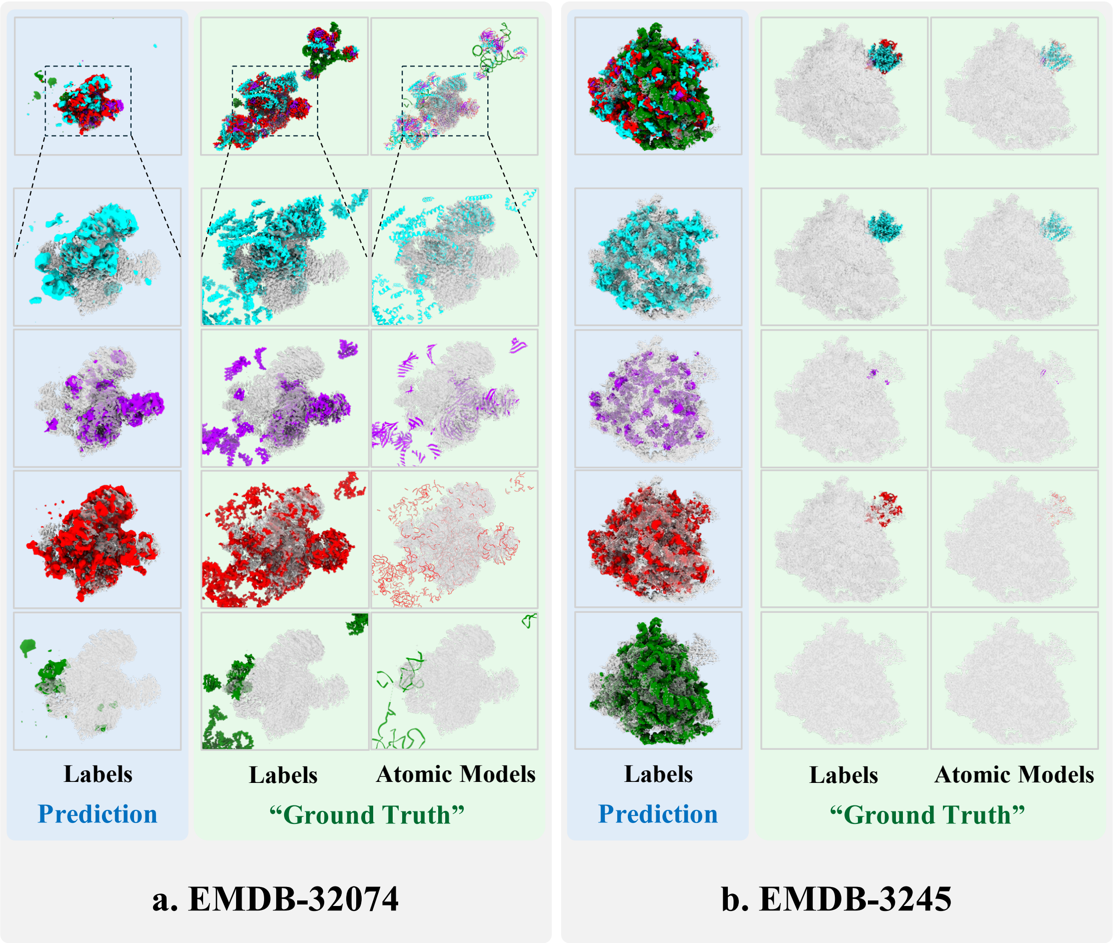

Fig. 4: Representative predictions from the U-net model trained on the experimental dataset, showing one example with low recall and another with low precision on the test set. Predicted labels—including composite outputs and separate annotations for helix, sheet, coil, and RNA—are shown alongside the corresponding “ground truth”, derived from manually built atomic models in the PDB. Blue panels show predictions; green panels show “ground truth”. cryoEM maps are rendered as transparent overlays. Color code: cyan (helix), purple (sheet), red (coil), green (RNA). **a,** Example from EMDB-32074, where substantial portions of the ground truth atomic model are built without supporting cryoEM map density. The U-net model correctly abstains from predicting in these areas, leading to low recall. **b**, Example from EMDB-3245, where large regions of the cryoEM map remain unmodeled in the ground truth. The U-net model predicts plausible structures in these areas, resulting in low precision when evaluated against the incomplete ground truth.

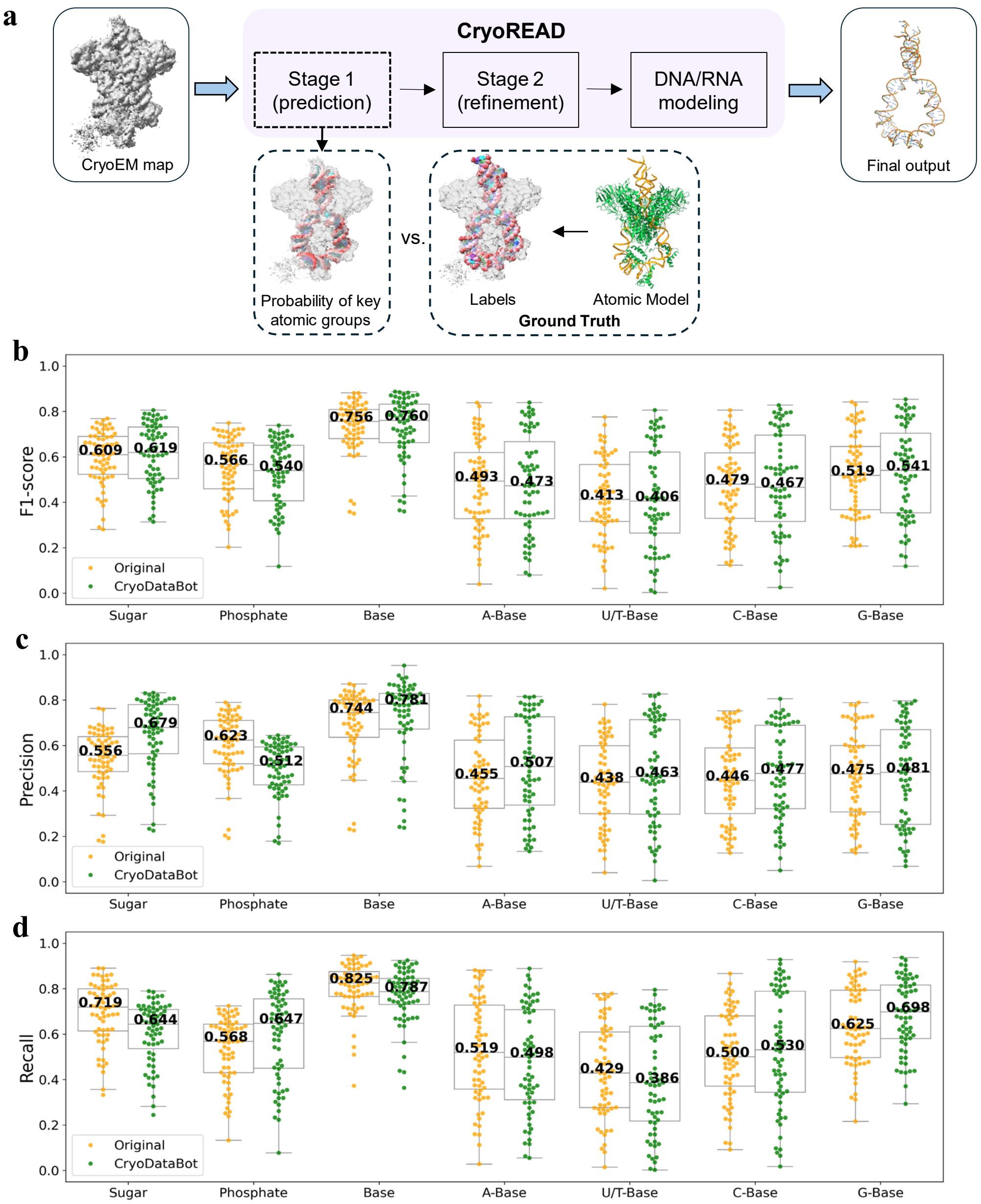

Fig. 5: Comparative evaluation of prediction performance between the original and retrained CryoREAD Stage 1 models. a, Overview of the CryoREAD workflow. The input is a cryoEM map and the final out put is an automatically built DNA or RNA atomic model. Stage 1 of CryoREAD identifies and classifies key atomic groups (sugar, phosphate, base, and base types), and its predictions are compared against reference labels derived from the deposited atomic model (example entry: EMD-7480, PDB ID: 6CIJ). **b,** F1 scores for each structural label, calculated on an independent test set of n = 63 cryoEM maps. Results are shown for the original CryoREAD model (yellow) and the retrained version using the CryoDataBot-genrated dataset (green). Median values are indicated within each box plot. **c-d**, Same as **b**, but for precision (**c**) and recall (**d**) metrics.

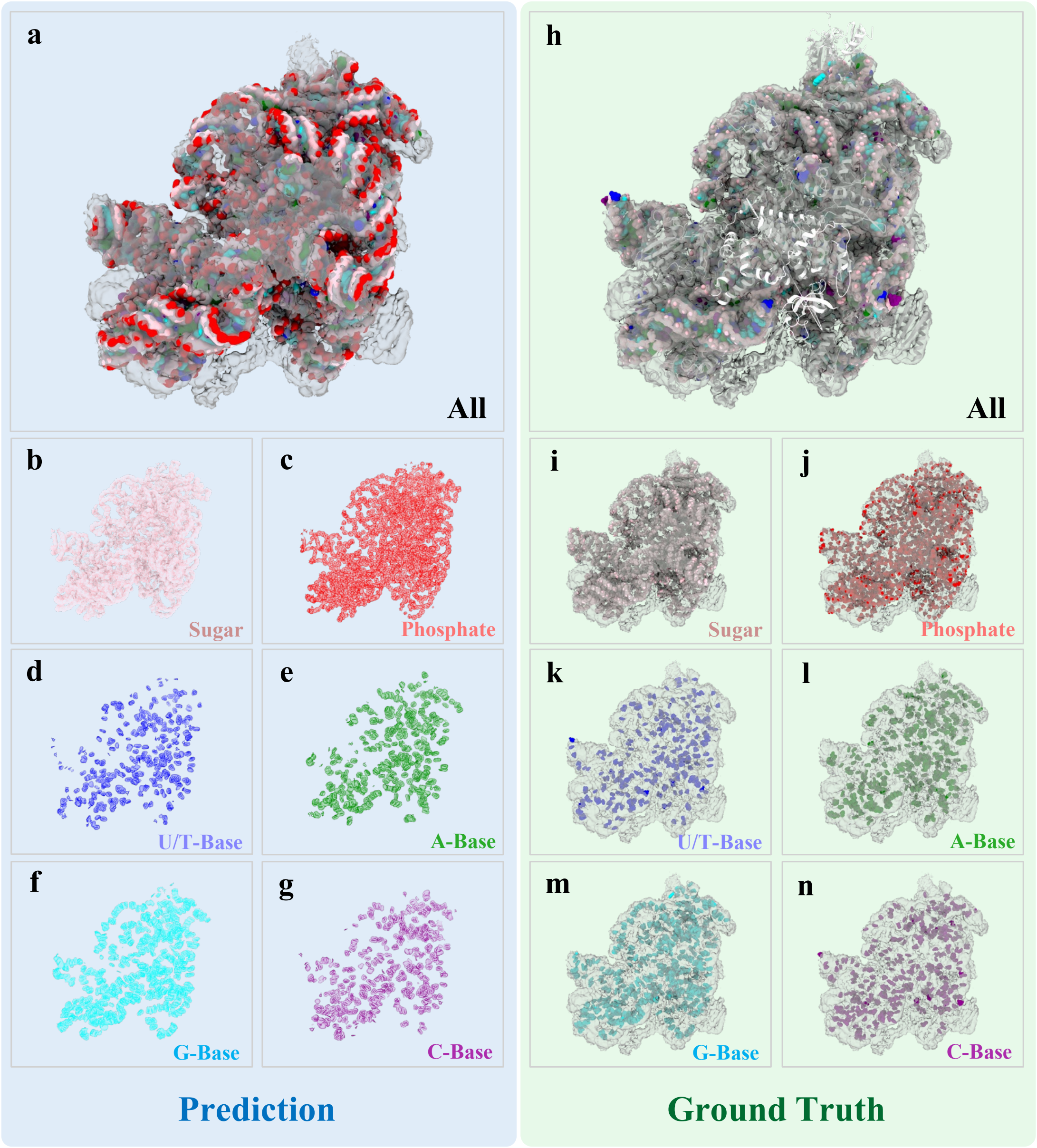

Fig. 6: Representative example (EMD-3532) of a test-set prediction from the retrained CryoREAD model. CryoREAD predictions (regions with probability > 0.4) are shown in the blue panels, while the corresponding ground truth atomic models are shown in the green panels. Different colors indicate different atomic groups: pink for sugar, red for phosphate, blue for U/T, green for A, light blue for G, purple for C, and white for protein (shown only in panel **h**). **a**, Composite prediction (colored volumes) overlaid with the original cryoEM map (gray). **b-g**. Individual predictions (transparent volumes) for each atomic group, overlaid with the ground truth atomic model (ball-and-stick representation). **h**. Ground truth atomic model, with RNA shown as spheres and protein as a cartoon, overlaid with the cryoEM map (gray). **i-n,** Ground truth representations for individual atomic groups (shown as spheres), overlaid with the original cryoEM map (gray).

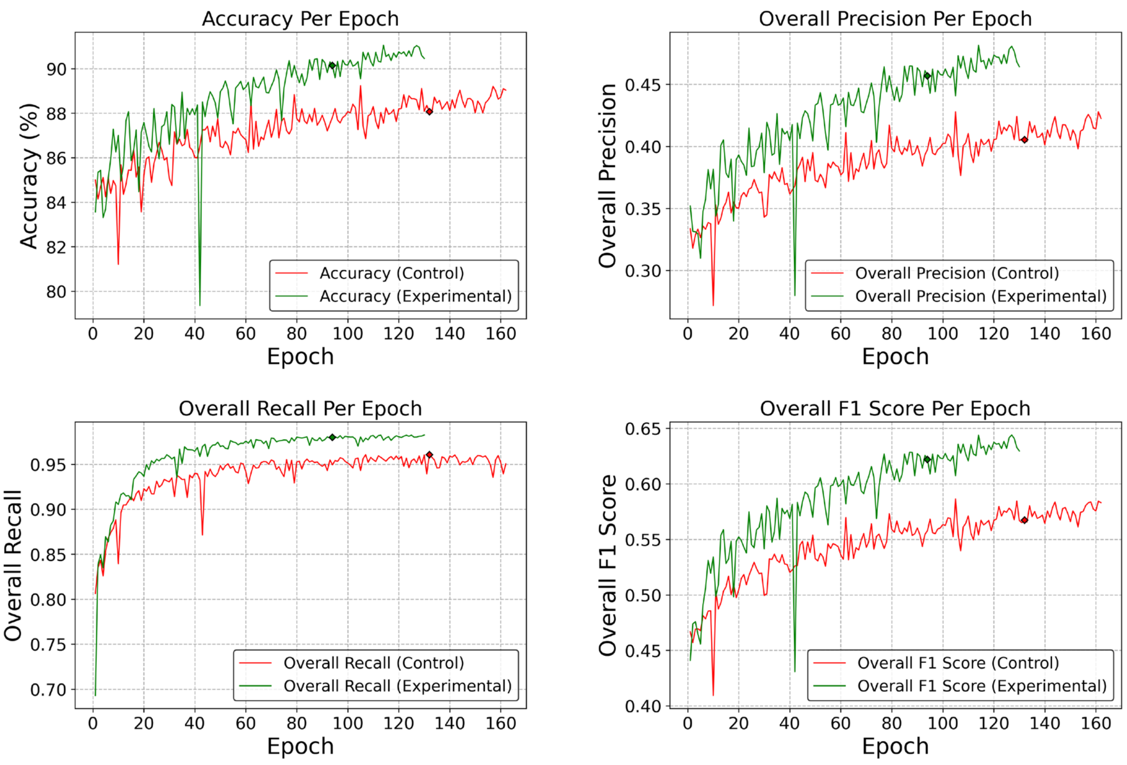

Supplementary Fig. 1 Training dynamics of U-Net models training on control and experimental datasets. Evolution of overall accuracy, precision, recall, and F1 score on the validation set during training of the control model (red) and the experimental model (green). Epochs corresponding to the best performance are marked by diamonds at epoch 132 (control) and epoch 94 (experimental). The experimental model reached higher validation metrics in fewer epochs, indicating improved training efficiency.

The following are titles and legends for the 4 Supplementary Tables in Excel files:

Supplementary Table 1. Metadata of the experimental dataset. (please click the blue URL link at the upper right corner above each table to see the full contents of each separate Excel file)

This Excel file comprises multiple tabs, which include the final curated entries from the experimental dataset as well as the entries excluded at each stage of quality control. The columns labeled "title," "resolution," "emdb_id," "fitted_pdbs," "xref_UNIPROTKB," "xref_ALPHAFOLD," "Q-score," "atom_inclusion," and "recommended_contour_level" correspond to the entry title, EMDB ID, fitted PDB identifiers, cross-references to the Universal Protein Knowledgebase (UniProtKB) and AlphaFold, Q-score, atom inclusion, and the recommended contour level, respectively, as retrieved from the EMDB (see Methods for details). Additionally, the columns labeled "vof," "dice_coefficient," "CC_mask," "CC_volume," "CC_peaks," and "CC_box" represent the calculated VOF score, Dice-like score, and various correlation coefficient measures, respectively, as detailed in the Methods section.

Supplementary Table 2. Metadata of the control dataset. (please click the blue URL link at the upper right corner above each table to see the full contents of each separate Excel file)

Similar to Supplementary Table 1, but for the control dataset.

Supplementary Table 3. Metadata of the raw dataset. (please click the blue URL link at the upper right corner above each table to see the full contents of each separate Excel file)

Similar to Supplementary Table 1, but for the raw dataset.

Supplementary Table 4. Metadata of the test dataset. (please click the blue URL link at the upper right corner above each table to see the full contents of each separate Excel file)

Similar to Supplementary Table 1, this file contains two tabs, titled "for_U-net" and "for_CryoREAD," which provide metadata for the test dataset used to evaluate U-net and CryoREAD, respectively.

MS Excel file for Supplementary Table 1

Click here to access/download
Supplementary Material
TableS1-Experimental_dataset_metadata.xlsx

MS Excel file for Supplementary Table 2

Click here to access/download
Supplementary Material
TableS2-Control_dataset_metadata.xlsx

MS Excel file for Supplementary Table 3

Click here to access/download
Supplementary Material
TableS3-Raw_dataset_metadata.xlsx

MS Excel file for Supplementary Table 4

Click here to access/download

Supplementary Material

TableS4-Test_dataset_metadata.xlsx

UNIVERSITY OF CALIFORNIA, LOS ANGELES

BERKELEY • DAVIS • IRVINE • LOS ANGELES • MERCED • RIVERSIDE • SAN DIEGO • SAN FRANCISCO

UCLA

SANTA BARBARA • SANTA CRUZ

Z. Hong Zhou, PhD
PROFESSOR & DIRECTOR
Electron Imaging Center for NanoSystems
California NanoSystems Institute (CNSI)
Los Angeles, CA 90095-7151
http://EICN.ucla.edu and http://www.mimg.ucla.edu/

DEPARTMENT OF MICROBIOLOGY, IMMUNOLOGY & MOLECULAR GENETICS
DAVID GEFFEN SCHOOL OF MEDICINE AT UCLA
Box 951594
Los Angeles, CA 90095-1594
Courier delivery: 570 Westwood Plaza, UCLA Building 114, CNSI 5511C, Los Angeles, CA 90095
EMAIL: Hong.Zhou@UCLA.edu; PHONE: 310-694-7527

July 7, 2025

Editors
GigaScience

Dear Editors and Editorial Staff,

I am pleased to submit our manuscript, "**CryoDataBot: a pipeline to curate cryoEM datasets for AI-driven structural biology,**" for consideration as an Article in **GigaScience**. Authored by Qibo Xu *et al.*, this work introduces a novel, user-friendly pipeline for generating standardized, high-quality cryogenic electron microscopy (cryoEM) datasets, specifically designed to facilitate AI-driven structural biology research.

CryoDataBot fills a critical need by automating data retrieval, curation, preprocessing, and quality control form public datasets, enabling researchers to easily create reproducible and customizable cryoEM datasets. Its modular design supports diverse downstream applications, such as training AI models—U-Net and CryoREAD—as well as benchmarking and validation, as demonstrated by our evaluations. The tool and datasets are publicly available, promoting transparency and accelerating AI-driven structural modeling research. This work strongly aligns with GigaScience's mission to advance open science, large-scale data analysis, and reproducibility.

The manuscript has not been published elsewhere, nor is it under consideration by another journal. All authors have approved the submission and declare no competing interests. The CryoDataBot code is available under an MIT license at https://github.com/t00shadow/CryoDataBot.

Thank you for your time and attention. Please direct correspondence to me at Hong.Zhou@UCLA.edu or 310-694-7527.

Sincerely,

Z. Hong Zhou, PhD, Professor and founding director of Electron Imaging Center for Nanosystems at UCLA

Suggested Reviewers:

Min Xu, Associate professor at Carnegie Mellon University, AI tools development for cryoEM
mxu1@andrew.cmu.edu

Alberto Bartesaghi, Professor of Duke University, AI-based tool development for cryoEM and cryoET
alberto@cs.duke.edu

Ellen Zhong, Assistant professor at Princeton University, AI-based data processing software
zhonge@cs.princeton.edu

Jose-Maria Carazo, Group leader at CNB-CSIC, Spain, World authority of cryoEM software development
carazo@cnb.uam.es

Qiangfeng Cliff Zhang, Professor at Tsinghua University, Beijing, China, CryoNet.Fold developer
qczhang@tsinghua.edu.cn

Steven Ludtke, Professor at Baylor College of Medicine, EMAN developer and machine learning
sludtke@bcm.edu

Bridget Carragher, Research Director, Chan Zuckerberg Imaging Institute, cryoEM automation and software development
bridget.carragher@czii.org

Mingxu Hu, Assistant professor/group leader at SMART, cryoPROS developer
humingxu@smart.org.cn

Toshio Moriya, SPHIRE developer
toshio.moriya@kek.jp

Alexis Rohou, Genetec group leader and developer of *CTFFIND4* and *cisTEM*
rohou.alexis@gene.com

Joey Davis, Associate Professor at MIT, developer of *SIREn* for structural heterogeneity analysis
jhdavis@mit.edu

We respectfully request to exclude **Jianyi Yang** (yangjy@sdu.edu.cn) and **Sheng-You Huang** (huangsy@hust.edu.cn) as reviewers of this work due to conflict of interest.
