# Supplementary material for: CryoDataBot: a pipeline to curate cryoEM datasets for AI-driven structural biology
Source: Gigascience. 2025 Oct 22;14:giaf127. doi: 10.1093/gigascience/giaf127 (PMC12596181; doi:10.1093/gigascience/giaf127)
Supplement: giaf127_Authors_Response_To_Reviewer_Comments_Original_Submission [file giaf127_authors_response_to_reviewer_comments_original_submission.pdf]

Author's Response To Reviewer Comments

(For improved formatting and readability, we kindly refer reviewers to the PDF version of our response.)

Summary: We thank all three reviewers for carefully reviewing our paper and for the valuable suggestions. The main revisions include additional benchmarking on a G protein dataset with ablation of Q-score thresholds, addition of confusion matrices with extended discussion on the precision–recall tradeoff, and expanded analysis and implementation details of quality control processes.
As you can see below, we have incorporated all suggestions made by the reviewers and highlighted the related text changes in the revised manuscript. To facilitate your perusal of the following itemized statement, we have copied the reviewers' comments in black and our responses are shown in blue.

Reviewer #1 (Remarks to the Author):
The authors introduce CryoDataBot, a GUI-driven pipeline for automatically curating cryo EM map / model pairs into machine learning-ready datasets. The study is timely and addresses a real bottleneck in AI driven atomic model building. The manuscript is generally well written and benchmarking experiments (U Net and CryoREAD retraining). Nevertheless, several conceptual and presentation issues should be resolved before the work is suitable for publication:

1. All quantitative tests focus on ribosome maps in the 3-4 Å range. Because ribosomes are unusually large and RNA rich, it is unclear whether the curation criteria (especially Q score ≥ 0.4 and VOF ≥ 0.82) generalise to smaller or lower resolution particles. Please include at least one additional macromolecule class (e.g. membrane proteins or spliceosomes) or justify why the current benchmark is sufficient.
Response: We conducted additional benchmarking using a G protein dataset. During training dataset construction, we applied the same curation criteria used for ribosomes—specifically, a Q-score ≥ 0.4 and a similarity score ≥ 0.7. Given the smaller size of the G protein dataset (126 entries compared to 942 ribosome entries), we adjusted the MMF filter (VOF) to 0.65 to ensure a sufficient number of entries for training. The training results confirmed that these settings remained effective, supporting their use as reasonable default thresholds. This analysis is described in the added section Robustness Check: Benchmarking on G protein Dataset.

2. The manuscript adopts fixed thresholds (Q score 0.4; 70 % similarity; VOF 0.82) yet does not show how sensitive downstream model performance is to these values. A short ablation (e.g. sweep the Q score from 0.3-0.6) would help readers reuse the tool sensibly.
Response: In response to the reviewers' suggestion, we conducted a short ablation study by sweeping the Q-score from 0.3 to 0.5 during G protein dataset construction, and trained U-Net models on the resulting datasets to assess performance changes. A precision–recall tradeoff was observed across thresholds, with Q-score ≥ 0.4 yielding the highest F1 score. (Lines 369-387)
We recommend that users apply the default settings (Q-score ≥ 0.4) as a starting point, then adjust the filtering thresholds—either more permissive or more restrictive—based on the intended application of the model, and perform a grid search to optimize predictive performance. (Lines 388-394)

3. Table 1 claims CryoDataBot "addresses omissions" of Cryo2StructData, but no quantitative head to head benchmarking is provided (e.g. train the same U Net on Cryo2StructData). Please add such a comparison or temper the claim.
Response: We have revised the caption in Table 1 by removing the statement that CryoDataBot "addresses omissions" in Cryo2StructData, and have tempered our comparison to avoid implying direct superiority. The updated wording reflects a more neutral description of each dataset's features without making unsupported claims.

4. For voxel wise classification, F1 scores are affected by severe class imbalance (Nothing ≫ Helix/Sheet/Coil/RNA). Report per class support (number of positive voxels) and consider complementary instance level or backbone trace metrics.
Response: We have added the confusion matrices to Fig. S4 and Fig. 7c to report per-class support and illustrate how voxel counts influence performance (Lines 250–254, 378–387). The differing results between

the control/raw and experimental training models are primarily driven by changes in the number of true positives and false positives. The lower recall observed in the experimental model reflects a slight reduction in true positives. However, despite this decrease, the more substantial reduction in false positives—enabled by cleaner and more conservative training data—results in higher precision and, consequently, an overall improvement in F1 score.

5. In Fig. 4 the authors show that poor recall/precision partly stems from erroneous deposited models. Quantify how often this occurs across the 18 map test set and discuss implications for automated QC inside CryoDataBot.
Response: Outlier points falling outside the interquartile range in Figure 3c-e correspond to cases similar to those illustrated in Figure 4, where poor ground truth quality affects model evaluation. To address this, we added clarifications in Lines 261–263 and 281–286, emphasizing that CryoDataBot's Q-score and MMF filters serve as automated quality control mechanisms. The excluded entries during dataset construction were structurally inconsistent.

6. The authors note improved precision but slightly reduced recall in CryoDataBot-trained models. This is explained, but strategies to mitigate this tradeoff are not discussed. Could ensemble learning, soft labeling, or multi-resolution data alleviate the recall drop?
Response: As noted in Lines 249–254 and 383–387, the observed drop in recall can be attributed to the reduced sample size and diversity caused by stringent filtering. The simplest way to mitigate this effect is to relax the quality control filters. By appropriately adjusting these filters, the model can achieve a better balance—maintaining high precision while minimizing recall loss (Fig. 7). Therefore, we recommend performing a grid search over different thresholds when maximizing overall performance is necessary (Lines 392–394).
More broadly, we agree with the reviewer that several established strategies could further help improve recall in classification tasks. These include adjusting decision thresholds to favor sensitivity, applying data augmentation to increase sample diversity, and incorporating soft labeling to retain low-confidence regions. Additionally, techniques such as multi-resolution input, class rebalancing, and ensemble learning have been shown to enhance recall by capturing broader structural variation and reducing overfitting. While these methods are not yet implemented in the current version of CryoDataBot, they represent promising directions for future optimization depending on downstream application needs.

Reviewer #2 (Remarks to the Author):
This paper discusses CryoDataBot, which creates cryoEM datasets for the use of training with the abilities to filter out based on redundancy, MMF and other user defined parameters. Here are some comments:
1. The data labeling just has helix, sheet, coil, and RNA. The labeling should also consider DNA and other structures.
Response: The data labeling scheme also includes DNA, and we have incorporated an indirect approach to annotate additional structural elements beyond the predefined categories. To clarify this, we have updated the Methods section (GUI part, Lines 611 and 615–617) of the manuscript accordingly.

2. The introduction of a Volume Overlap Fraction (VOF) score to validate map-model fitness (MMF) is a novel method to assess global alignment. However, VOF relies on summing and binarizing 2D projections which may have limitations. It is not clear how sensitive the VOF score is to the binarization process or how it handles complex, non-globular shapes. The paper would be strengthened if the authors could provide more justification for this specific metric over other global 3D correlation scores. An analysis of specific examples of map-model pairs that were discarded by the VOF score but not by the Q-score would be informative.
Response: The primary reason that we employ the VOF score instead of other global 3D correlation metrics is computational efficiency during large-scale dataset construction. A voxel-by-voxel comparison between cryoEM maps and pseudo-maps (generated from atomic models) requires substantial memory bandwidth and runtime, particularly for large cryoEM volumes. A second reason is conceptual: while the Q-score already highlights local density features (and may overfit to them), in the MMF stage we aim to emphasize global structural coherence, which the VOF score captures more effectively.
By binarizing and projecting maps onto six planes (orthogonal to the x-, y-, and z-axes, as well as to the three major body diagonals), the VOF score remains robust even for complex, non-globular shapes, as demonstrated in Fig. S1. This figure also provides examples with low VOF but high Q-scores, illustrating

cases where local density alignment does not translate into meaningful global fit. Notably, most entries discarded during MMF validation in the G protein dataset fall into this category of non-globular shapes. These clarifications, together with Fig. S1, have been incorporated into both the main text and the Methods section (Lines 107–111 and 505–512). Overall, VOF and Q-score are complementary: VOF emphasizes global consistency while Q-score captures local details, and their combination enhances the robustness of quality control for map-model fitness.

3. The authors acknowledge the trade-off between higher precision and lower recall that results from overly stringent filtering. While increased precision clearly benefits tasks like model refinement, the resulting reduced recall could be a significant hinder de novo modeling which is dependent upon capturing the entirety of a structure, even with lower confidence. This point could be elaborated on. Is this an area for future work, .e.g. developing pre-configured filtering settings for various downstream tasks, like a Precision vs. Recall bias setting? This might increase utility based on application.
Response: We have added the confusion matrices for the models in Fig. S4 and Fig. 7c, together with a detailed explanation of how the stringency of filtering influences recall and precision (Lines 249–254 and 383–387). The simplest way to increase precision is to make the filtering more stringent, and to improve recall is to relax the filtering. While there may exist an optimal threshold that maximizes the F1 score, this threshold cannot be predetermined because it depends on the specific characteristics of a user's dataset. For this reason, it is challenging to design universal pre-configured filtering settings.
To further illustrate this point, we conducted a short ablation study by sweeping the Q-score threshold from 0.3 to 0.5 during G protein dataset construction. The results showed that Q-score ≥ 0.4 yielded the highest F1 score for the trained U-Net model on this dataset (Lines 369–387). Based on this, we recommend that users apply default settings (Q-score ≥ 0.4) as a starting point, then adjust the filtering thresholds—either more permissive or more stringent—as needed to optimize predictive performance (Lines 388–394).

4. The retraining of CryoREAD is a practical validation of the pipeline's utility for RNA modeling, however the experimental dataset used is exclusively from ribosomes. Ribosomes were selected because they contain both protein and RNA and are abundant in the EMDB but they may not represent the full diversity of RNA structures. The authors rightly note that training set composition affects performance. It would be helpful to further discuss the potential shortcomings of an exclusively ribosome-based training set and possible impact to the retrained CryoREAD model's use validating other classes of RNA.
Response: We thank the reviewer for the insightful suggestion. We have added a paragraph (Lines 343–350) in the CryoREAD section discussing the implications of training on a ribosome-only dataset. As shown in Fig. S5, the retrained CryoREAD performs consistently better on ribosome cases (38/63 test maps) than on other RNA/DNA classes (25 maps), highlighting reduced generalizability compared to the original CryoREAD trained on more diverse structures (Fig. S6). At the same time, ribosome-only training enhances predictive performance specifically for ribosome structures, underscoring the critical role of training dataset composition in determining model applicability and performance.

5. The author should consider benchmarking on the other SOTA protein-RNA-DNA modeling tools. Right now it is only benchmarked on their own CryoREAD which is just a RNA/DNA modeling tool.
Response: We thank the reviewer for this valuable suggestion. Our focus in this work is on validating the effectiveness of CryoDataBot as a data pipeline rather than benchmarking across all existing modeling tools. To this end, we selected U-Net as our primary benchmark because it is one of the most widely adopted architectures in cryo-EM AI-based methods for biomolecular structure modeling, underlying diverse applications such as particle picking1, post-processing2, 3D Reconstruction and 3D Refinement3, automated model building4–6, structure fitting7, and validation8. Demonstrating CryoDataBot's benefits on U-Net therefore illustrates its broad utility across multiple downstream tasks.
In addition, we included CryoREAD as a case study to further show applicability to RNA/DNA modeling. While we did not benchmark against other SOTA protein–RNA–DNA modeling tools in this study, we agree this is an important future direction and plan to extend CryoDataBot evaluation to additional frameworks as these become more widely available. We also welcome future opportunities for collaboration or comparative studies with other tools to further highlight the generality and value of CryoDataBot.

6. I tried installing CryoDataBot and looks like it requires python version 3.8 or higher but isn't specified anywhere in the paper or the site.
Response: We have updated the GitHub README to specify the required Python version (≥3.10).

7. Many references and citations are off and wrong.
Response: We appreciate the reviewer's attention to detail. We have carefully checked all references and citations throughout the manuscript and corrected any inaccuracies or other issues. We thank the reviewer for pointing this out, and we have ensured that the revised version adheres to proper citation standards.

Reviewer #3 (Remarks to the Author):
The paper presents a flexible, integrated framework for filtering and generating customizable cryo-EM training datasets. It builds upon previously available strategies for preparing cryo-EM datasets for AI-based methods, extending them with a user-friendly interface that allows researchers to enter query parameters, interact directly with the Electron Microscopy Data Bank (EMDB), extract and parse relevant metadata, apply quality control measures, and retrieve associated structural data (cryo-EM maps and atomic models).

1. While the manuscript improves upon Cryo2StructData and similar data pipelines used in ModelAngelo/DeepTracer, the innovation claim would be strengthened by a deeper technical comparison, for example quantifying the performance impact of each quality control step in isolation. Some filtering and preprocessing concepts (e.g., voxel resampling, redundancy handling) are not entirely new, so a more explicit discussion of how CryoDataBot's implementations differ from prior work and why these differences matter would improve the manuscript. I do not think its challenging to change the resampling or the grid division parameter on the scripts provided by Cryo2StructData github repo or scripts available in ModelAngelo github repo.

Response: We agree that voxel resampling and grid division are not novel components, and we do not claim innovation in these aspects. As noted, such parameters are indeed adjustable in existing pipelines such as Cryo2StructData and ModelAngelo. Instead, our contributions focus on the design and implementation of effective quality control steps—specifically Q-score filtering, redundancy filtering, and VOF filtering. These components are central to CryoDataBot's ability to construct high-quality datasets and are described in detail in both the Pipeline and Methods sections.
To substantiate the impact of these quality control steps, we conducted three quantitative evaluations:
(1)Dataset-level comparison: We benchmarked datasets with and without quality control, as described in the section "Construction of Benchmarking Datasets Using CryoDataBot." Notably, datasets from Cryo2StructData and DeepTracer are equivalent to the raw dataset in our study. Figures 2 and S2 illustrate the influence of quality control steps—primarily Q-score and redundancy filtering—while Figure S1 demonstrates the role of VOF filtering in ensuring global consistency between cryoEM maps and model pairs.
(2) Model-level comparison (ribosome): We trained U-Net models on ribosome datasets with and without quality control and compared their performance. Figures 3, 4, and S4 quantitatively show improved training efficiency and higher precision when using datasets processed with quality control steps.
(3) Model-level comparison (G protein): We performed an additional benchmark using G protein datasets, evaluating model performance across different Q-score thresholds to assess the effect of varying filtering stringency. Figures 7 and S7 demonstrate improved training efficiency with quality-controlled datasets, and provide a detailed analysis of the influence of different Q-score thresholds.
Together, these tests demonstrate that CryoDataBot's quality control steps are not only practical but also effective in improving downstream model performance.

2. The benchmarking is mainly limited to ribosome datasets. While this choice is understandable for demonstration purposes, the generalizability to other macromolecules (e.g., membrane proteins, small complexes) is not shown. This can include a small-scale test on a different class of structures (e.g., protein's C-alpha positions, backbone atom position or amino acid type prediction (more difficult one) could strengthen the claim of broad applicability. Since the technical innovation limited, this can help to improve the paper.
Response: In response to the reviewer's suggestion, we conducted an additional benchmarking test using G proteins, which are notably smaller in size compared to ribosomes. We trained U-Net models to identify C-alpha positions, thereby extending the evaluation to a distinct macromolecular class. The analysis is presented in the newly added results section titled "Robustness Check: Benchmarking on G Protein Dataset."
The results show that CryoDataBot-processed G protein datasets improve training efficiency and precision

relative to unfiltered datasets (Figs. 7 and S7), consistent with the findings from ribosome benchmarks. These tests demonstrate that CryoDataBot can be applied beyond ribosomes and remains effective for other classes of macromolecules.

3. The authors state that CryoDataBot ensures reproducibility and provides datasets for AI-method benchmarking. However, EMDB entries can be updated over time (e.g., through reprocessing, resolution improvements, model re-fitting, or correction of atomic coordinates). In my opinion, in the strict sense, reproducibility (producing identical datasets) depends on versioning of EMDB/PDB entries. Without version locking, CryoDataBot ensures procedural reproducibility but not data immutability. The manuscript should either explain how reproducibility is maintained (e.g., version control, archived snapshots) or clarify that reproducibility refers to the workflow, not necessarily the exact dataset content, unless version dataset are provided, as done in Cryo2StructData.
Response: We agree with reviewer about "reproducibility" and now indicated in the revised manuscript that the reproducibility refers to the workflow reproducibility in Line 64. We also added Lines 451–452, clarifying the exact date and time the dataset was fetched, to help reproduce our results.
Some other concerns:

4. The "Generating Structural Labels" section has missing technical details. Please provide more information on how the labels are generated, including labeling radius selection, and how ambiguities are resolved if any encountered. A suggestions on how the user should determine the radius and also the grid size (64^3 or other) would be beneficial.
Response: We have added more technical details in the Methods section regarding how structural labels are generated. While our original text briefly mentioned how ambiguities are resolved, we have now expanded this explanation. Additionally, we included guidance on how users can determine appropriate labeling radius and grid size, rather than relying on the default values (Lines 537–548).

5. The manuscript states on the adaptive density normalization part: "This method is more flexible and removes more noise than the fixed-threshold approaches commonly used in prior studies." What does noise and signals mean here? - there is a separate body of AI-based works developed for reducing noise such as DeepEMhancer, EMReady to name few. Any metric to support this claim?
Response: Here, noise refers to low-intensity background values that do not correspond to molecular features, while signal denotes high-density regions representing structural information. Because the noise–signal boundary varies across EM maps, fixed thresholds (e.g., zero) used in prior studies may overlook map-specific variability. To address this, we developed an Adaptive Map Normalization strategy that uses map-specific denoising threshold computed from the metadata field recommended_contour_level. This ensures consistent removal of low-intensity noise—values between zero and the map-specific threshold—and improves normalization robustness across datasets (Lines 483–503).
While methods like DeepEMhancer and EMReady offer advanced enhancement, our goal was to provide a fast and lightweight preprocessing pipeline focused on efficiency, so we did not incorporate these methods into our workflow.

6. The manuscript states: "To assess dataset redundancy, we analyzed structural similarity between entries based on InterPro (IPR) domain annotations." Is this a new approach introduced here, or an established practice? How does it compare with sequence-based similarity measures? Or Structure-based similarity such as Foldseek?
Response: The use of IPR domain annotations to assess dataset redundancy is a novel approach introduced in this work. While not yet a standard practice, it builds on the principle of local alignment and offers domain-level resolution that is functionally meaningful.
To complement this, we have added a sequence-based redundancy analysis using BLASTp (Lines 201–205). As shown in Fig. S2, the BLASTp results are consistent with the IPR-based analysis: the Raw and Control datasets exhibit comparable levels of redundancy, whereas the Experimental dataset shows a substantial reduction. (We note that chain-level homology can oversimplify structure alignment and may include regions that are not structurally or functionally conserved.)

7. The statement "underscoring the dataset's superior quality and informativeness" is strong. Is it possible to provide more concrete, quantitative evidence to support this, ideally beyond the U-Net training metrics.?
Response: We have removed this statement from the manuscript. In addition, we have revised other

sections to reduce overly strong language. Please let us know if you find any further instances that should be qualified.

8. Is there a case where there is multiple PDB IDs for the cryo-EM density map? If so how is a specific atomic model chosen in such case?
Response: Yes. If multiple PDB IDs are associated with an EMDB entry, only the first PDB ID listed in EMDB is retrieved as the metadata (Lines 446–447). We do not further distinguish between these entries, since in most cases their structural differences are minimal and quality variation is negligible, leading to little or no impact on the outcomes of subsequent quality control (QC) processes.
Future versions of CryoDataBot could allow users to specify alternative PDB IDs if desired.

REFERENCES
1. Gyawali, R., Dhakal, A., Wang, L., and Cheng, J. (2024). CryoSegNet: accurate cryo-EM protein particle picking by integrating the foundational AI image segmentation model and attention-gated U-Net. Brief. Bioinform. 25, bbae282.
2. Sanchez-Garcia, R., Gomez-Blanco, J., Cuervo, A., Carazo, J.M., Sorzano, C.O.S., and Vargas, J. (2021). DeepEMhancer: a deep learning solution for cryo-EM volume post-processing. Commun. Biol. 4, 874.
3. Liu, Y.-T., Fan, H., Hu, J.J., and Zhou, Z.H. (2025). Overcoming the preferred-orientation problem in cryo-EM with self-supervised deep learning. Nat. Methods 22, 113–123.
4. Pfab, J., Phan, N.M., and Si, D. (2021). DeepTracer for fast de novo cryo-EM protein structure modeling and special studies on CoV-related complexes. Proc. Natl. Acad. Sci. 118, e2017525118. https://doi.org/10.1073/pnas.2017525118.
5. Wang, X., Terashi, G., and Kihara, D. (2023). CryoREAD: de novo structure modeling for nucleic acids in cryo-EM maps using deep learning. Nat. Methods, 1–9. https://doi.org/10.1038/s41592-023-02032-5.
6. Terashi, G., Wang, X., Prasad, D., Nakamura, T., and Kihara, D. (2024). DeepMainmast: integrated protocol of protein structure modeling for cryo-EM with deep learning and structure prediction. Nat. Methods 21, 122–131.
7. Wang, X., Zhu, H., Terashi, G., Taluja, M., and Kihara, D. (2024). DiffModeler: large macromolecular structure modeling for cryo-EM maps using a diffusion model. Nat. Methods 21, 2307–2317.
8. Terashi, G., Wang, X., Maddhuri Venkata Subramaniya, S.R., Tesmer, J.J., and Kihara, D. (2022). Residue-wise local quality estimation for protein models from cryo-EM maps. Nat. Methods 19, 1116–1125.

em Editorial Manager®

Cookie Preference Center

We use cookies which are necessary to make our site work. We may also use additional cookies to analyze, improve and personalize our content and your digital experience. For more information, see our Cookie Policy.

You may choose not to allow some types of cookies. However, blocking some types may impact your experience of our site and the services we are able to offer. See the different category headings below to find out more or change your settings.

Allow all

Manage Consent Preferences

Strictly Necessary Cookies

Always active

These cookies are necessary for the website to function and cannot be switched off in our systems. They are usually only set in response to actions made by you which amount to a request for services, such as setting your privacy preferences, logging in or filling in forms. You can set your browser to block or alert you about these cookies, but some parts of the site will not then work.

Functional Cookies

These cookies enable the website to provide enhanced functionality and personalisation. They may be set by us or by third party providers whose services we have added to our pages. If you do not allow these cookies then some or all of these services may not function properly.

Performance Cookies

These cookies allow us to count visits and traffic sources so we can measure and improve the performance of our site. They help us to know which pages are the most and least popular and see how visitors move around the site.

Targeting Cookies

These cookies may be set through our site by our advertising partners. They may be used by those companies to build a profile of your interests and show you relevant adverts on other sites. If you do not allow these cookies, you will experience less targeted advertising.

Cookie List

Clear

Apply Cancel

Consent Leg.Interest

Confirm my choices

Powered by onetrus
