# Supplementary material for: CryoDataBot: a pipeline to curate cryoEM datasets for AI-driven structural biology
Source: Gigascience. 2025 Oct 22;14:giaf127. doi: 10.1093/gigascience/giaf127 (PMC12596181; doi:10.1093/gigascience/giaf127)
Supplement: giaf127_Reviewer_1_Report_Original_Submission — Ashwin Dhakal -- 7/27/2025 [file giaf127_reviewer_1_report_original_submission.pdf]

Reviewer Report

Title: CryoDataBot: a pipeline to curate cryoEM datasets for AI-driven structural biology

Version: Original Submission **Date:** 7/27/2025

Reviewer name: Ashwin Dhakal

Reviewer Comments to Author:

The authors introduce CryoDataBot, a GUIâ€�driven pipeline for automatically curating cryo EM map / model pairs into machine learning-ready datasets. The study is timely and addresses a real bottleneck in AI driven atomic model building. The manuscript is generally well written and benchmarking experiments (U Net and CryoREAD retraining). Nevertheless, several conceptual and presentation issues should be resolved before the work is suitable for publication:1 All quantitative tests focus on ribosome maps in the 3-4â€¯Ã… range. Because ribosomes are unusually large and RNA rich, it is unclear whether the curation criteria (especially Q scoreâ€¯â‰¥â€¯0.4 and VOFâ€¯â‰¥â€¯0.82) generalise to smaller or lower resolution particles. Please include at least one additional macromolecule class (e.g. membrane proteins or spliceosomes) or justify why the current benchmark is sufficient.2 The manuscript adopts fixed thresholds (Q scoreâ€¯0.4; 70â€¯% similarity; VOFâ€¯0.82) yet does not show how sensitive downstream model performance is to these values. A short ablation (e.g. sweep the Q score from 0.3-0.6) would help readers reuse the tool sensibly.3 Tableâ€¯1 claims CryoDataBot "addresses omissions" of Cryo2StructData, but no quantitative head to head benchmarking is provided (e.g. train the same U Net on Cryo2StructData). Please add such a comparison or temper the claim.4 For voxel wise classification, F1 scores are affected by severe class imbalance (Nothingâ€¯â‰«â€¯Helix/Sheet/Coil/RNA). Report per class support (number of positive voxels) and consider complementary instance level or backbone trace metrics.5 In Fig.â€¯4 the authors show that poor recall/precision partly stems from erroneous deposited models. Quantify how often this occurs across the 18 map test set and discuss implications for automated QC inside CryoDataBot.6 The authors note improved precision but slightly reduced recall in CryoDataBot-trained models. This is explained, but strategies to mitigate this tradeoff are not discussed. Could ensemble learning, soft labeling, or multi-resolution data alleviate the recall drop?

Level of Interest

Please indicate how interesting you found the manuscript: Choose an item.

Quality of Written English

Please indicate the quality of language in the manuscript: Choose an item.

Declaration of Competing Interests

Please complete a declaration of competing interests, considering the following questions:

- Have you in the past five years received reimbursements, fees, funding, or salary from an organisation that may in any way gain or lose financially from the publication of this manuscript, either now or in the future?
- Do you hold any stocks or shares in an organisation that may in any way gain or lose financially from the publication of this manuscript, either now or in the future?
- Do you hold or are you currently applying for any patents relating to the content of the manuscript?
- Have you received reimbursements, fees, funding, or salary from an organization that holds or has applied for patents relating to the content of the manuscript?
- Do you have any other financial competing interests?
- Do you have any non-financial competing interests in relation to this paper?

If you can answer no to all of the above, write 'I declare that I have no competing interests' below. If your reply is yes to any, please give details below.

I declare that I have no competing interests.

I agree to the open peer review policy of the journal. I understand that my name will be included on my report to the authors and, if the manuscript is accepted for publication, my named report including any attachments I upload will be posted on the website along with the authors' responses. I agree for my report to be made available under an Open Access Creative Commons CC-BY license (http://creativecommons.org/licenses/by/4.0/). I understand that any comments which I do not wish to be included in my named report can be included as confidential comments to the editors, which will not be published.

Choose an item.

To further support our reviewers, we have joined with Publons, where you can gain additional credit to further highlight your hard work (see: https://publons.com/journal/530/gigascience). On publication of this paper, your review will be automatically added to Publons, you can then choose whether or not to claim your Publons credit. I understand this statement.

Yes Choose an item.
