# Supplementary material for: CryoDataBot: a pipeline to curate cryoEM datasets for AI-driven structural biology
Source: Gigascience. 2025 Oct 22;14:giaf127. doi: 10.1093/gigascience/giaf127 (PMC12596181; doi:10.1093/gigascience/giaf127)
Supplement: giaf127_Reviewer_2_Report_Original_Submission — Dong Si -- 8/7/2025 [file giaf127_reviewer_2_report_original_submission.pdf]

Reviewer Report

Title: CryoDataBot: a pipeline to curate cryoEM datasets for AI-driven structural biology

Version: Original Submission **Date:** 8/7/2025

Reviewer name: Dong Si

Reviewer Comments to Author:

This paper discusses CryoDataBot, which creates cryoEM datasets for the use of training with the abilities to filter out based on redundancy, MMF and other user defined parameters. Here are some comments:- The data labeling just has helix, sheet, coil, and RNA. The labeling should also consider DNA and other structures.- The introduction of a Volume Overlap Fraction (VOF) score to validate map-model fitness (MMF) is a novel method to assess global alignment. However, VOF relies on summing and binarizing 2D projections which may have limitations. It is not clear how sensitive the VOF score is to the binarization process or how it handles complex, non-globular shapes. The paper would be strengthened if the authors could provide more justification for this specific metric over other global 3D correlation scores. An analysis of specific examples of map-model pairs that were discarded by the VOF score but not by the Q-score would be informative.- The authors acknowledge the trade-off between higher precision and lower recall that results from overly stringent filtering. While increased precision clearly benefits tasks like model refinement, the resulting reduced recall could be a significant hinder de novo modeling which is dependent upon capturing the entirety of a structure, even with lower confidence. This point could be elaborated on. Is this an area for future work, .e.g. developing pre-configured filtering settings for various downstream tasks, like a Precision vs. Recall bias setting? This might increase utility based on application.- The retraining of CryoREAD is a practical validation of the pipeline's utility for RNA modeling, however the experimental dataset used is exclusively from ribosomes. Ribosomes were selected because they contain bothprotein and RNA and are abundant in the EMDB but they may not represent the full diversity of RNA structures. The authors rightly note that training set composition affects performance. It would be helpful to further discuss the potential shortcomings of an exclusively ribosome-based training set and possible impact to the retrained CryoREAD model's use validating other classes of RNA.- The author should consider benchmarking on the other SOTA protein-RNA-DNA modeling tools. Right now it is only benchmarked on their own CryoREAD which is just a RNA/DNA modeling tool.- I tried installing CryoDataBot and looks like it requires python version 3.8 or higher but isn't specified anywhere in the paper or the site.- Many references and citations are off and wrong.

Level of Interest

Please indicate how interesting you found the manuscript: Choose an item.

Quality of Written English

Please indicate the quality of language in the manuscript: Choose an item.

Declaration of Competing Interests

Please complete a declaration of competing interests, considering the following questions:

- Have you in the past five years received reimbursements, fees, funding, or salary from an organisation that may in any way gain or lose financially from the publication of this manuscript, either now or in the future?
- Do you hold any stocks or shares in an organisation that may in any way gain or lose financially from the publication of this manuscript, either now or in the future?
- Do you hold or are you currently applying for any patents relating to the content of the manuscript?
- Have you received reimbursements, fees, funding, or salary from an organization that holds or has applied for patents relating to the content of the manuscript?
- Do you have any other financial competing interests?
- Do you have any non-financial competing interests in relation to this paper?

If you can answer no to all of the above, write 'I declare that I have no competing interests' below. If your reply is yes to any, please give details below.

I declare that I have no competing interests

I agree to the open peer review policy of the journal. I understand that my name will be included on my report to the authors and, if the manuscript is accepted for publication, my named report including any attachments I upload will be posted on the website along with the authors' responses. I agree for my report to be made available under an Open Access Creative Commons CC-BY license (http://creativecommons.org/licenses/by/4.0/). I understand that any comments which I do not wish to be included in my named report can be included as confidential comments to the editors, which will not be published.

Choose an item.

To further support our reviewers, we have joined with Publons, where you can gain additional credit to further highlight your hard work (see: https://publons.com/journal/530/gigascience). On publication of this paper, your review will be automatically added to Publons, you can then choose whether or not to claim your Publons credit. I understand this statement.

Yes Choose an item.
