# Supplementary material for: CryoDataBot: a pipeline to curate cryoEM datasets for AI-driven structural biology
Source: Gigascience. 2025 Oct 22;14:giaf127. doi: 10.1093/gigascience/giaf127 (PMC12596181; doi:10.1093/gigascience/giaf127)
Supplement: giaf127_Reviewer_3_Report_Original_Submission — Nabin Giri -- 8/7/2025 [file giaf127_reviewer_3_report_original_submission.pdf]

Reviewer Report

Title: CryoDataBot: a pipeline to curate cryoEM datasets for AI-driven structural biology

Version: Original Submission **Date:** 8/7/2025

Reviewer name: Nabin Giri

Reviewer Comments to Author:

The paper presents a flexible, integrated framework for filtering and generating customizable cryo-EM training datasets. It builds upon previously available strategies for preparing cryo-EM datasets for AI-based methods, extending them with a user-friendly interface that allows researchers to enter query parameters, interact directly with the Electron Microscopy Data Bank (EMDB), extract and parse relevant metadata, apply quality control measures, and retrieve associated structural data (cryo-EM maps and atomic models).While the manuscript improves upon Cryo2StructData and similar data pipelines used in ModelAngelo/DeepTracer, the innovation claim would be strengthened by a deeper technical comparison, for example quantifying the performance impact of each quality control step in isolation. Some filtering and preprocessing concepts (e.g., voxel resampling, redundancy handling) are not entirely new, so a more explicit discussion of how CryoDataBot's implementations differ from prior work and why these differences matter would improve the manuscript. I do not think its challenging to change the resampling or the grid division parameter on the scripts provided by Cryo2StructData github repo or scripts available in ModelAngelo github repo.The benchmarking is mainly limited to ribosome datasets. While this choice is understandable for demonstration purposes, the generalizability to other macromolecules (e.g., membrane proteins, small complexes) is not shown. This can include a small-scale test on a different class of structures (e.g., protein's C-alpha positions, backbone atom position or amino acid type prediction (more difficult one) could strengthen the claim of broad applicability. Since the technical innovation limited, this can help to improve the paper.The authors state that CryoDataBot ensures reproducibility and provides datasets for AI-method benchmarking. However, EMDB entries can be updated over time (e.g., through reprocessing, resolution improvements, model re-fitting, or correction of atomic coordinates). In my opinion, in the strict sense, reproducibility (producing identical datasets) depends on versioning of EMDB/PDB entries. Without version locking, CryoDataBot ensures procedural reproducibility but not data immutability. The manuscript should either explain how reproducibility is maintained (e.g., version control, archived snapshots) or clarify that reproducibility refers to the workflow, not necessarily the exact dataset content, unless version dataset are provided, as done in Cryo2StructData.Some other concerns:(1) The "Generating Structural Labels" section has missing technical details. Please provide more information on how the labels are generated, including labeling radius selection, and how ambiguities are resolved if any encountered. A suggestions on how the user should determine the radius and also the grid size (64^3 or other) would be beneficial.(2) The manuscript states on the adaptive density normalization part : "This method is more flexible and removes more noise than the fixed-threshold approaches commonly used in prior studies." What does noise and signals mean here? - there is a separate body of AI-based works developed for reducing noise such as DeepEMhancer, EMReady to name few. Any metric to support this claim?(3) The manuscript

states: "To assess dataset redundancy, we analyzed structural similarity between entries based on InterPro (IPR) domain annotations." Is this a new approach introduced here, or an established practice? How does it compare with sequence-based similarity measures? Or Structure-based similarity such as Foldseek?(4) The statement "underscoring the dataset's superior quality and informativeness" is strong. Is it possible to provide more concrete, quantitative evidence to support this, ideally beyond the U-Net training metrics.?(5) Is there a case where there is multiple PDB IDs for the cryo-EM density map? If so how is a specific atomic model chosen in such case?

Level of Interest

Please indicate how interesting you found the manuscript: Choose an item.

Quality of Written English

Please indicate the quality of language in the manuscript: Choose an item.

Declaration of Competing Interests

Please complete a declaration of competing interests, considering the following questions:

- Have you in the past five years received reimbursements, fees, funding, or salary from an organisation that may in any way gain or lose financially from the publication of this manuscript, either now or in the future?
- Do you hold any stocks or shares in an organisation that may in any way gain or lose financially from the publication of this manuscript, either now or in the future?
- Do you hold or are you currently applying for any patents relating to the content of the manuscript?
- Have you received reimbursements, fees, funding, or salary from an organization that holds or has applied for patents relating to the content of the manuscript?
- Do you have any other financial competing interests?
- Do you have any non-financial competing interests in relation to this paper?

If you can answer no to all of the above, write 'I declare that I have no competing interests' below. If your reply is yes to any, please give details below.

I declare that I have no competing interest

I agree to the open peer review policy of the journal. I understand that my name will be included on my report to the authors and, if the manuscript is accepted for publication, my named report including any attachments I upload will be posted on the website along with the authors' responses. I agree for my report to be made available under an Open Access Creative Commons CC-BY license (http://creativecommons.org/licenses/by/4.0/). I understand that any comments which I do not wish to

be included in my named report can be included as confidential comments to the editors, which will not be published.

Choose an item.

To further support our reviewers, we have joined with Publons, where you can gain additional credit to further highlight your hard work (see: https://publons.com/journal/530/gigascience). On publication of this paper, your review will be automatically added to Publons, you can then choose whether or not to claim your Publons credit. I understand this statement.

Yes Choose an item.
